# Strain-Level Variation and Diverse Host Bacterial Responses in Episymbiotic Saccharibacteria

**DOI:** 10.1128/msystems.01488-21

**Published:** 2022-03-28

**Authors:** Jie Nie, Daniel R. Utter, Kristopher A. Kerns, Eleanor I. Lamont, Erik L. Hendrickson, Jett Liu, Tingxi Wu, Xuesong He, Jeffrey McLean, Batbileg Bor

**Affiliations:** a Department of Cariology and Endodontology, Peking University School of Stomatology, Beijing, China; b Department of Organismic and Evolutionary Biology, Harvard University, Cambridge, Massachusetts, USA; c Department of Periodontics, University of Washington, Seattle, Washington, USA; d The Forsyth Institute, Cambridge, Massachusetts, USA; e Department of Oral Medicine, Infection and Immunity, Harvard School of Dental Medicine, Boston, Massachusetts, USA; University of British Columbia

**Keywords:** human, mouth, microbiota, parasite, culture technique, genomics, bacteria, candidate phyla radiation, Saccharibacteria, TM7, human

## Abstract

Saccharibacteria (TM7), which are obligate episymbionts growing on the surface of host bacteria, may play an important role in oral disease, such as periodontitis (1, 2). As TM7 is a newly cultured lineage of bacteria, its research is limited by the small number of isolated representatives relative to the number of TM7 genomes assembled from culture-independent studies (3–5). A comprehensive view of both TM7 taxa and TM7 strain-level variations remains opaque. In this study, we expanded our previously developed TM7 baiting method into using many host bacteria in parallel, which allowed us to obtain 37 TM7 strains from the human oral cavity. These strains were further classified into low-enrichment (LE, *n* = 24) and high-enrichment (HE, *n* = 13) groups based on their proficiency at propagating on host bacteria. Of the 13 HE strains, 10 belong to “*Candidatus* Nanosynbacter sp.” strain HMT-352 (human microbial taxon) (6), enabling us to explore both the phenotypic and genomic strain variations within a single TM7 species. We show that TM7 HMT-352 strains exhibit a diverse host range and varied growth dynamics during the establishment of their episymbiotic relationship with host bacteria. Furthermore, despite HMT-352 strains sharing a majority of their genes, we identified several gene clusters that may play a pivotal role in host affinity. More importantly, our comparative analyses also provide TM7 gene candidates associated with strain-level phenotypic variation that may be important for episymbiotic interactions with host bacteria.

**IMPORTANCE** Candidate phylum radiation (CPR) bacteria comprise a poorly understood phylum that is estimated to encompass ∼26% of all diversity of domain bacteria. Among CPR bacteria, the Saccharibacteria lineage (TM7) is of particular interest, as it is found in high abundance in the mammal microbiome and has been associated with oral disease. While many CPR genomes, TM7 included, have been acquired through culture-independent methods, only a small number of representatives have been isolated. Such isolated representatives, however, shed light on the physiology, pathogenesis, and episymbiotic interactions of TM7. Combined with genomic analyses, experiments involving isolated representatives can distinguish phylogenetic to phenotypic discrepancies and better identify genes of importance. In this study, we utilized multiple host bacteria in parallel to isolate TM7 bacteria and examined strain-level variation in TM7 to reveal key genes that may drive TM7-host interactions. Our findings accentuate that broad phylogenetic characterization of CPR is the next step in understanding these bacteria.

## INTRODUCTION

The candidate phylum radiation (CPR) is a large monophyletic radiation of bacteria (>73 phyla) with reduced genomes, currently referred to as the superphylum Patescibacteria (7, 8). Among these, Saccharibacteria (formerly known as TM7) contain the first cultured representative, “*Candidatus* Nanosynbacter lyticus” TM7x, from the human oral cavity and is associated with the human microbiome (9). In recent years, cultivation of additional TM7 strains from human and environmental sources has been achieved (3–5, 10, 11). All known TM7 strains are obligate episymbionts that grow on the surface of host bacteria (12, 13). TM7, with an ultrasmall cell size (200 to 500 nm) and a reduced genome (<1 Mb), lack the capacity to synthesize many essential molecules, such as amino acids, vitamins, and cell wall precursors (14).

TM7x, as an obligate episymbiont, is typically cocultured with the host *Schaalia odontolytica* strain XH001 (previously known as Actinomyces odontolyticus) (15). When TM7x infects XH001 via cell surface attachment, it initially induces a “growth-crash” phenotype in XH001 by inhibiting cell growth and division (12). When TM7x is incubated with other host bacteria, some hosts display a susceptible phenotype (infected with TM7x) while others exhibit a nonsusceptible phenotype (cannot be infected with TM7x). Furthermore, susceptible hosts can demonstrate either “permissive” or “nonpermissive” phenotypes, i.e., some hosts experience the growth-crash phenomenon while others do not (16). While current knowledge of cultivatable TM7 strains is limited to the G1 clade of TM7, previous research has indicated that the TM7 host range is restricted to specific subsets of bacteria from the phylum *Actinobacteria* (16). Studying TM7 and their host interactions is pertinent to the fields of oral microbiology and medicine, as TM7 strains are recognized to be core members of the oral microbiome and are associated with periodontal disease (1, 2).

One of the major impediments in researching TM7 remains the limited number of isolated TM7 representatives, both in phylogenetic breadth and the number of strains per species (9, 17). To further study the phenotypic, physiological, genetic, and pathogenic characteristics of TM7, more isolates are required. Current methods of TM7 isolation include targeted enrichment approaches, reverse genomics, and a “baiting” method. The first isolated TM7 strain, TM7x, was isolated from SHI medium-cultivated saliva samples by using targeted enrichment approaches that leveraged the streptomycin resistance of TM7x (18). Cross et al. developed a reverse genomics approach and successfully isolated three different species of human oral TM7 (3). Based on the ability of free-floating TM7 cells to infect new host bacteria, we previously developed a novel host baiting method by filtering and concentrating TM7 cells prior to coculturing with candidate hosts (4). From these studies, it is clear that identification of proper candidate hosts is crucial in acquiring a stable TM7-host binary coculture.

Strain-level variation is a basic feature of bacteria that dictate their survival in diverse environmental niches (19, 20) and is a key factor in determining their physiology (21), pathogenesis (22, 23), and, for episymbionts, specific bacterium-host interactions (24). The limited isolated and characterized TM7 strains belong to different species or genera, and analyses have shown that they share similar general characteristics, such as ultrasmall cell size and a reduced genome, while differing in host preference (4). Although different strains belonging to the same species of TM7 have been previously isolated, nuances in TM7 biology and genome variation at a strain level remain largely understudied (5). As strains with almost identical genomes can exhibit differing physiological or pathogenic characteristics, understanding strain-level variation is a powerful tool in determining gene function. For example, in comparing the genomes of two Porphyromonas gingivalis strains with distinct immune-stimulatory capacities, Coats et al. revealed that the *fimB* allele was the key virulence gene. Further, a single polymorphism between the genomes resulted in the reduced immune-stimulatory capacity of one of the strains (25). Similarly, Biswas et al. found that in investigating a defective mutant strain of Lactobacillus rhamnosus, the *ftsH* gene plays an important role in the survival of L. rhamnosus within acidic environments such as the human oral cavity and gastrointestinal tract (26). Therefore, studying the strain-level variations among TM7 may be important in elucidating the genetic basis of their physiological functions, particularly since TM7 are a recently discovered group of episymbionts without a tractable genetic system developed.

Here, we expand upon our previous baiting technique to develop a high-throughput method for testing many host bacteria in parallel to isolate TM7 from the oral cavity. With our new method, we isolated and characterized multiple strains belonging to the same TM7 species, “*Candidatus* Nanosynbacter sp.” strain HMT-352 (HMT, human microbial taxon) (6). Additionally, we analyzed TM7 strain-level phenotypic differences, ranging from host range to their impact on hosts during establishment of episymbiosis. Furthermore, comparative genomic analyses were performed to investigate potential genetic bases of the differential phenotypic variation. Altogether, we found substantial physiological diversity among strains of TM7 HMT-352, with strains exhibiting differential host preferences despite seemingly minor genomic differences. Our findings underscore the impact of strain-level diversity on microbial ecology and highlight the importance of broad phylogenetic characterization of CPR bacteria.

## RESULTS

### High-throughput host bacterium testing and TM7 isolation.

**FIG 1:**
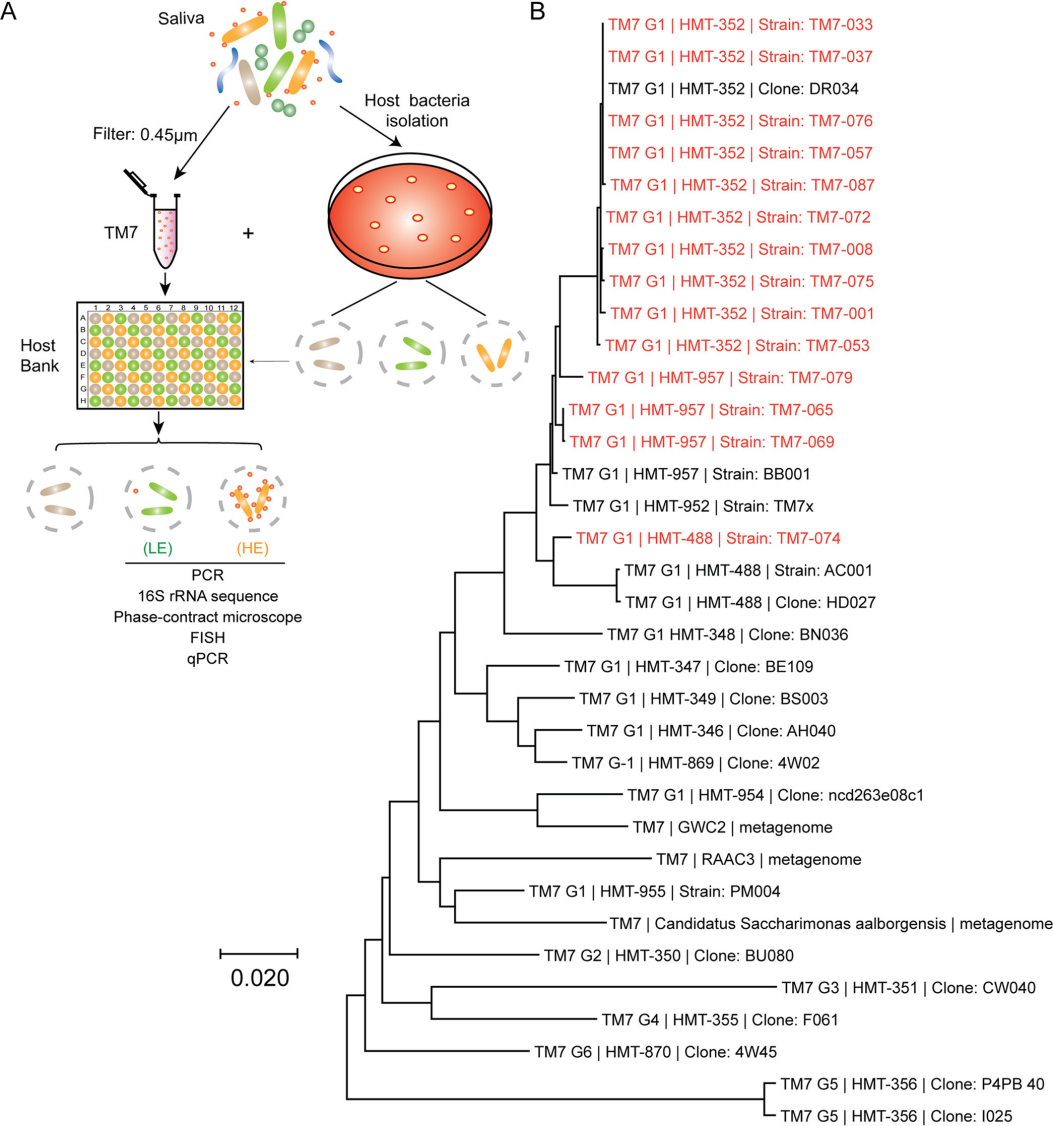
Isolation of TM7. (A) Workflow of TM7 isolation procedure that resulted in low- and high-enrichment groups (LE and HE). TM7 strains were isolated from saliva using a high-throughput 96-well plate host baiting technique. (B) Neighbor-joining tree of TM7 isolates in the HE group (highlighted in red). The tree was produced from aligned full-length 16S rRNA sequences of representative strains using MEGA X. Designations G1 to G6 are the TM7 class-level groups, as previously described. HMT numbers are shown according to the eHOMD.

Currently, all known TM7 are obligate episymbionts that grow on the surface of *Actinobacteria* members. To expand on this previously short list of basibionts, we cultured high-value host bacteria that may bait TM7 from the oral cavity (4). These basibionts (e.g., *Actinomyces*, *Schaalia*, *Cellulosimicrobium*, *Pseudopropionibacterium*, and *Corynebacterium*) were targeted based on their taxonomic relationship with previously known basibionts. Many new *Actinomyces* and *Schaalia* strains (*N* = 41) were cultivated from the healthy and periodontal human oral cavity using three independent blood agar selection plates (see Materials and Methods) (Fig. 1A). We named these new strains “JN” followed by a number (see Table S1 in the supplemental material). These putative basibionts were combined with preexisting strains to establish a 96-well plate system within which 76 different potential basibionts are held in separate wells.

10.1128/msystems.01488-21.5 TABLE S1 List of basibionts and their growth conditions. Detailed taxonomy, growth, and source information on basibionts that were tested. Download Table S1, XLSX file, 0.01 MB. Copyright © 2022 Nie et al. 2022 Nie et al. https://creativecommons.org/licenses/by/4.0/ This content is distributed under the terms of the Creative Commons Attribution 4.0 International license.

Using this basibiont plate as a screening platform, we exposed 16 periodontal filtered patient saliva samples to each unique basibiont, creating ∼1,200 unique coculture combinations (see Materials and Methods) (Fig. 1A). From this screen, we baited 38 TM7 strains onto different basibionts, and some TM7 strains were capable of infecting the same basibiont strain (Table 1B and Table S2). These cocultures were stably passaged over 4 times, and the presence of TM7 was verified by both PCR and microscopy techniques.

10.1128/msystems.01488-21.6 TABLE S2 Summary of all the TM7 strains that were isolated using high-throughput host bait method. Identity determined by 16S sequences. High abundance, HA; low abundance, LA. Download Table S2, XLSX file, 0.02 MB. Copyright © 2022 Nie et al. 2022 Nie et al. https://creativecommons.org/licenses/by/4.0/ This content is distributed under the terms of the Creative Commons Attribution 4.0 International license.

### Characterization of the cultured TM7 bacteria.

**FIG 2:**
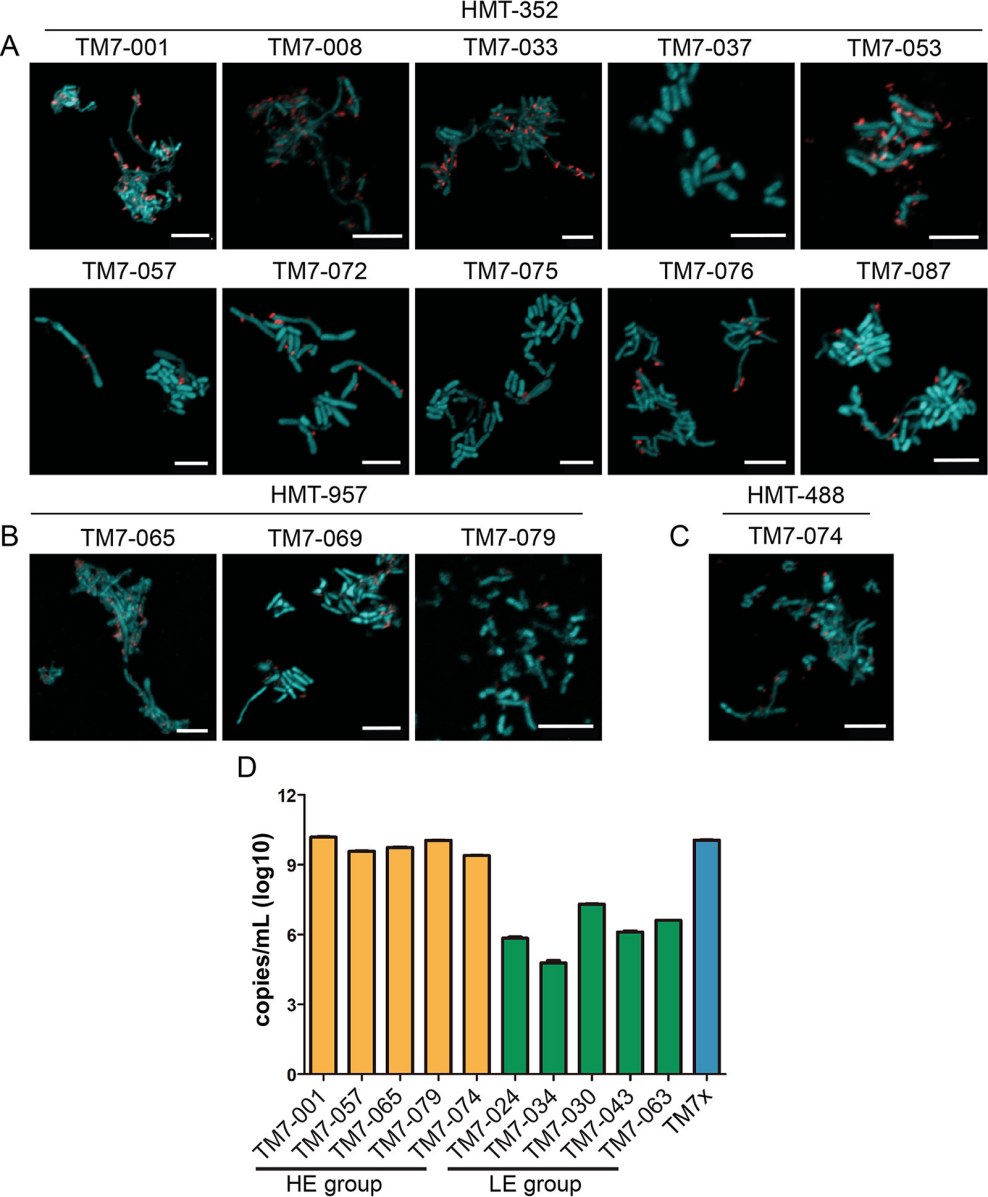
Different groups of isolated TM7. (A to C) FISH images of TM7 (red) and host bacteria (green) visualized by TM7-specific 16S rRNA probes. Basibionts were stained with Syto9 universal DNA dye. Only species in the HE group are shown by FISH. HMT-352 (A), HMT-957 (B), and HMT-488 (C) are illustrated by representative images. Scale bar, 5 μm. (D) qPCR reveals that copy numbers of TM7 16S per mL in the HE group (yellow bars) were ∼1,000 times greater than those in the LE group (green bars). In contrast, previously characterized TM7x is shown as the blue bar. Error bars indicate standard deviations.

Upon closer examination of the isolated TM7 cocultures, two distinct phenotypes emerged: cocultures containing large amounts of TM7 (high enrichment, HE), and cocultures with small amounts of TM7 (low enrichment, LE) (Fig. 1A and B and Table S2). The enrichment differences were clearly discernible by phase-contrast and fluorescence *in situ* hybridization (FISH) imaging. TM7 were visually abundant in the HE group (Fig. 2A to C) but not in the LE group (Fig. S1). Despite rarely detecting TM7 by microscopy in the LE group, the LE cocultures consistently tested positive for TM7 by PCR. When we quantified the amount of TM7 using a quantitative PCR (qPCR)-based method standardized to the copy number of a plasmid containing the TM7 16S sequence, the LE group contained 102 to 105 fewer TM7 than the HE group (Fig. 2D). This large discrepancy in TM7 abundance between the two groups is perhaps indicative that the LE TM7s are less compatible with their basibionts than the HE TM7s.

10.1128/msystems.01488-21.1 FIG S1 LE and HE TM7 and their basibionts. Phase-contrast (top row) and FISH (bottom row) images of LE and HE groups. FISH images of TM7 (red) and host bacteria (green) visualized by species-specific 16S rRNA probes. The LE cocultures were PCR positive for TM7 but displayed a weaker signal (see insets for the phase-contrast image). Download FIG S1, PDF file, 0.3 MB. Copyright © 2022 Nie et al. 2022 Nie et al. https://creativecommons.org/licenses/by/4.0/ This content is distributed under the terms of the Creative Commons Attribution 4.0 International license.

Within the HE group, the majority of the strains belonged to unnamed human microbial taxon (HMT) 352 (“*Ca*. Nanosynbacter spp.” [14]). A few were HMT-957 (“*Ca*. Nanosynbacter featherlites” [27]) and HMT-488 (“*Ca*. Nanosynbacter spp.”) strains (Fig. 1B and 2A to C). HMT designations are comparable to TM7 species classification (6). Isolating multiple strains of the same TM7 species capable of infecting different host bacteria is of interest given that our method utilized diverse basibionts as baits to isolate TM7 strains from multiple different patient samples. The 10 HMT-352 strains in the HE group, isolated from 6 different periodontal patients, were capable of infecting five different *Schaalia* basibionts (Table S2). Although initial saliva samples contained many different TM7 species, there are various potential reasons why HMT-352 was more readily isolated using our high-throughput method, spanning from specific culture conditions such as medium usage to potentially greater symbiotic plasticity among HMT-352 given our bait-based approach. Apparent through imaging, all strains isolated in this study grew on their basibionts in a manner similar to that of previously cocultured TM7 bacteria, and they did not show major differences in cell morphology under nutrition-replete conditions (Fig. 2A to C).

### Close examination of the host-range of TM7 strains within the same species.

**FIG 3:**
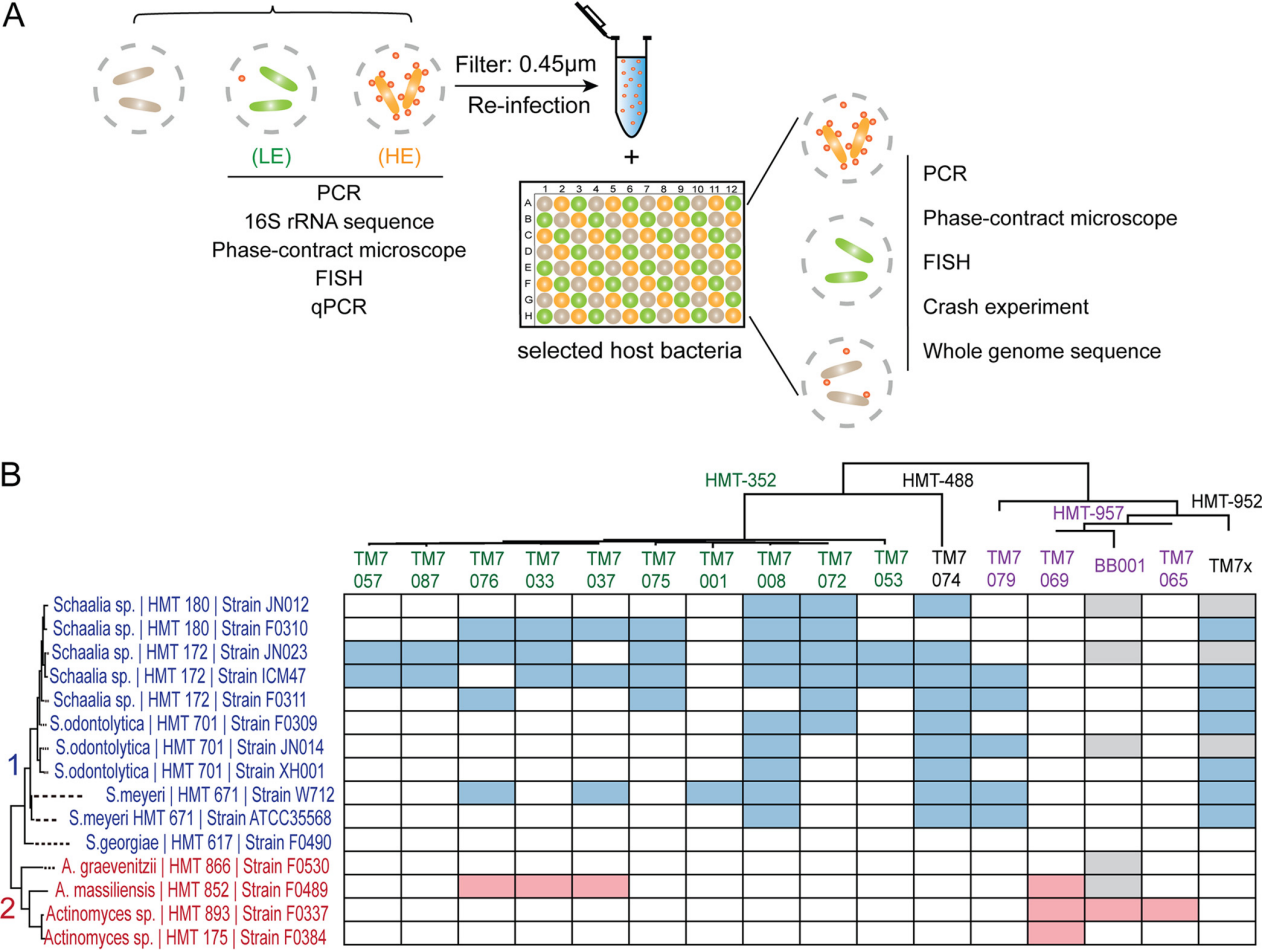
Host range in the HE group of TM7. (A) Schematic illustrating the various techniques used to determine the cultivated HE and LE groups of TM7 strains. (B) Host range of TM7 strains from the HE group. Blue (clade 1) and red (clade 2) blocks represent PCR-positive cocultures after various host bacteria (vertical) were infected with designated TM7 strains (horizontal) for 4 to 6 passages. White and gray blocks reflect PCR-negative and nontested host bacteria, respectively. The phylogenic tree was constructed using MEGA with ClustalW and neighbor-joining methods for full-length 16S rRNA sequences.

Cultivation of multiple strains belonging to the same TM7 species provided us with the opportunity to investigate both phenotypic and genomic variations within the same species of TM7 (Fig. 3A). To this end, we carefully tested the host range of these TM7 bacteria using previously established protocols (16). By isolating individual TM7 strains away from their original basibiont, we were able to infect a panel of *Schaalia* and *Actinomyces* spp. with each isolated TM7 strain (Fig. 3B). For each strain, we assessed its capacity for stable growth with individual basibiont species for an extended period of time (see Materials and Methods). Phylogenetically, the *Schaalia* and *Actinomyces* spp. largely grouped into two clades, 1 and 2 (12, 16). TM7 HMT-488 and -352 strains primarily grew on clade 1 (*Schaalia* spp.), similar to the previously described HMT-952 strain TM7x, while HMT-957 strains primarily grew on clade 2 (*Actinomyces* spp.), similar to the previously described BB001 strain, which also belongs to HMT-957 (Fig. 3B) (4). Surprisingly, there were several exceptions: HMT-352 strains TM7-033, TM7-037, and TM7-076 grew on the clade 2 F0489 basibiont. These three strains cluster together in the 16S gene taxonomy tree (Fig. 3B). TM7-079, unlike the other HMT-957 strains, grew on clade 1 *Schaalia* and is sequestered from the BB001, TM7-069, and TM7-065 clusters on the 16S tree.

Remarkably, although only one strain of HMT-352, TM7-072, was originally baited with basibiont ICM47, in our infection experiment, almost all HMT-352 strains, with the exception of TM7-001 and HMT-076, were able to grow with this basibiont, suggesting the broad ability of this basibiont to host HMT-352 strains (Fig. 3B). Alternatively, TM7 strains may be able to change their host preference rapidly. We also observed that TM7-008 and TM7-074 grew on the majority of the clade 1 basibionts, while basibiont JN023, closely related to strain ICM47, was also capable of hosting many of the HMT-352 strains. These results suggest that within the same species of TM7 bacteria, there are large phenotypic variations that include basibiont preference.

### Differential growth-crash responses induced by HMT-352 strains in basibionts.

**FIG 4:**
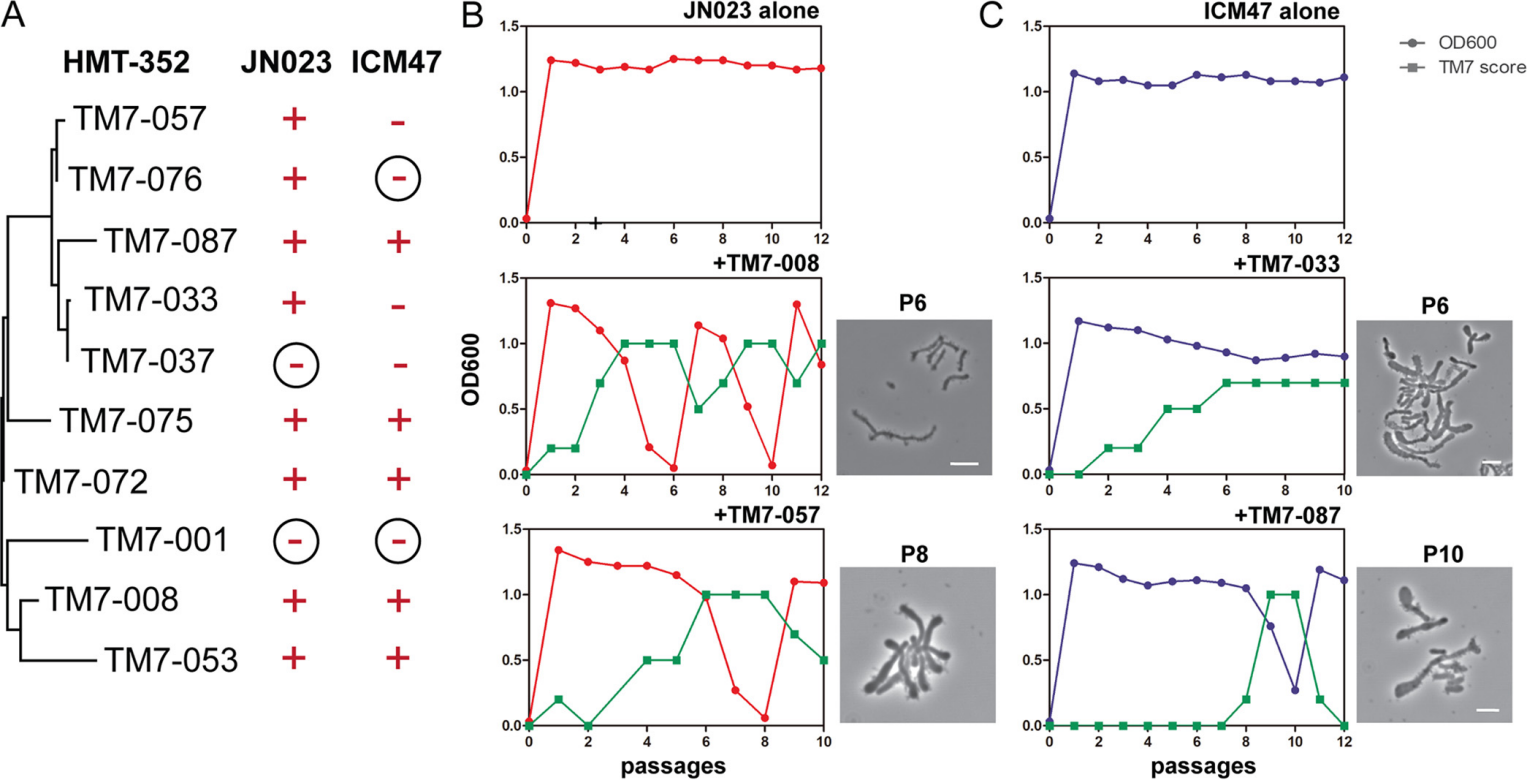
HMT-352 strains induce a growth-crash phenotype in their basibionts. (A) Two different host bacteria (JN023 and ICM47) were infected with 10 different HMT-352 TM7 strains. Subsequently, the growth-crash phenotype of the hosts was determined by cell density (OD_600_ measurements). Plus signs represent strains that crashed, while minus signs represent those that did not crash. Circled minus signs signify strains that were not susceptible to TM7 infection and did not display the growth-crash phenotype. (B and C) Representative growth-crash graphs for select TM7 strains from Fig. S2 and S3. The *x* axis represents the passage numbers across time, while the *y* axis represents both cell density measurements (OD_600_, circles) and TM7 scores (squares). Phase-contrast images show each culture during the indicated passages (scale bar, 2 μm).

We have previously compared various responses of basibionts to the same TM7 strain. However, the response of a single basibiont to different TM7 strains from the same species has not been tested. To investigate this, we assayed the growth-crash phenotype of the basibionts that the HMT-352 strains most commonly infected, JN023 and ICM47 (Fig. 3B). In this assay, we infected JN023 and ICM47 with different HMT-352 strains and then monitored cell optical density at 600 nm (OD_600_) and TM7 score (amount of TM7) during subsequent passages (see Materials and Methods). TM7-001 was the only strain that failed to infect either JN023 or ICM47 (Fig. 4A and Fig. S2 and S3). Every TM7 strain that successfully infected JN023 induced a severe growth-crash phenotype. As for ICM47, two TM7 strains did not infect ICM47, three strains infected ICM47 but did not induce the growth-crash phenotype, and five strains induced the growth-crash phenotype (Fig. 4A). These findings are consistent with our previous study of the TM7x strain, which infects ICM47 but does not induce the growth-crash phenotype (16). When a growth-crash occurred, we observed large variations in which some TM7 strains induced multiple sequential growth-crashes while other strains induced only a single growth-crash but at later passages (Fig. 4B and C and Fig. S2 and S3). Consistent with our previous studies, we microscopically observed an overwhelming number of TM7 infecting basibiont cells during the crash phase. The basibionts that did not crash showed sparse to no signs of TM7 infection (Fig. 4B and C). These findings were also reflected in the TM7 scores (Fig. S2 and S3).

10.1128/msystems.01488-21.2 FIG S2 Growth-crash phenotype in the JN023 basibiont that was infected with 10 different HMT-352 strains from the HE group. Expanded from Fig. 4 to illustrate each strain infection. The *x* axis represents passage numbers across time while the y axis represents both cell density measurements for host bacteria (OD_600_, circles) and TM7 scores (squares). See Materials and Methods for detailed experimental setup. Download FIG S2, PDF file, 1.9 MB. Copyright © 2022 Nie et al. 2022 Nie et al. https://creativecommons.org/licenses/by/4.0/ This content is distributed under the terms of the Creative Commons Attribution 4.0 International license.

10.1128/msystems.01488-21.3 FIG S3 Growth-crash phenotype in the ICM47 basibiont that was infected with 10 different HMT-352 strains from the HE group. Expanded from Fig. 4 to illustrate each strain infection. The *x* axis represents passage numbers across time, while the *y* axis represents both cell density measurements for host bacteria (OD_600_, circles) and TM7 scores (squares). See Materials and Methods for detailed experimental setup. Download FIG S3, PDF file, 1.6 MB. Copyright © 2022 Nie et al. 2022 Nie et al. https://creativecommons.org/licenses/by/4.0/ This content is distributed under the terms of the Creative Commons Attribution 4.0 International license.

### Genome analysis reveals substantial genetic heterogeneity among strains of HMT-352.

**FIG 5:**
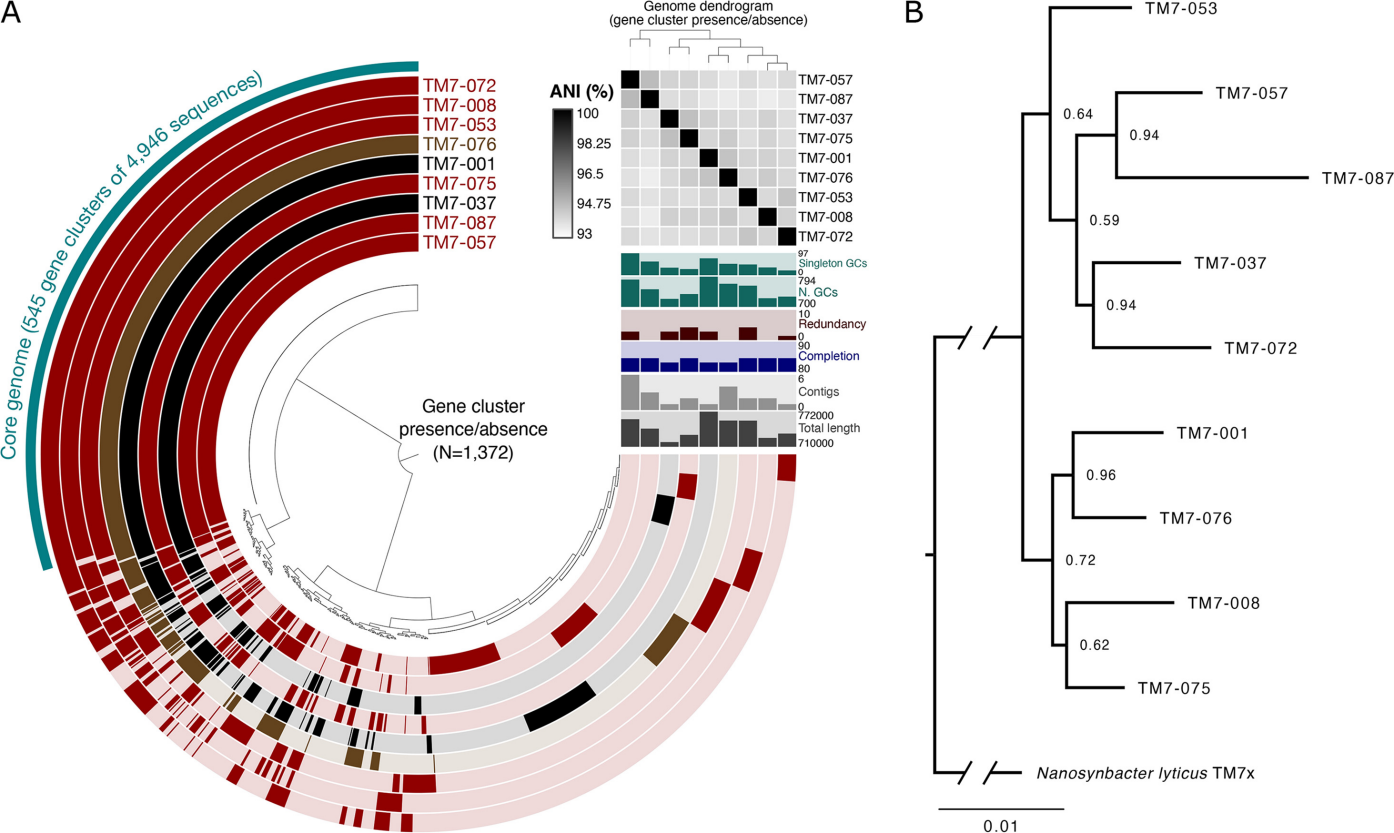
Comparative genomic and phylogenomic analyses of HMT-352 strains. (A) Pangenome showing the distribution of homologous gene clusters (tips of central radial dendrogram, *n* = 1,372) across the nine HMT-352 genomes (270° layers). Gene clusters and layers are both ordered by presence/absence. Layers are colored in to mark gene clusters found in that genome or left with a translucent background if the gene cluster is absent from that genome. Layers are colored according to the strain’s host phenotype: red, crashed both JN023 and ICM47; black, crashed neither; brown, crashed one. Relevant statistics for each genome (length, number of contigs, completion and redundancy, number of gene clusters, and number of singleton gene clusters) are shown in vertical bar charts extending from the 3 o’clock portion of the figure. The black and white heatmap shows pairwise average nucleotide identity (ANI) for all alignable fractions. (B) Phylogenomic tree constructed with 60 concatenated core proteins from the nine genomes shown in panel A with TM7x (HMT-952) included as an outgroup. Node labels represent bootstrap support.

We sequenced the 10 HMT-352 strains (TM7-001, TM7-008, TM7-037, TM7-053, TM7-057, TM7-072, TM7-075, TM7-076, TM7-087, and TM7-033) using Illumina short-read technology to assemble high-quality draft genomes (Fig. 5A). Genomes ranged from 718 kbp to 771 kbp and consisted of 1 to 6 contigs. All genomes were less than 5% redundant and between 83 and 85% complete, a completion percentage typical for complete genomes from obligate symbionts that lack universal genes found in free-living bacteria (28, 29). Further, each genome contained between 732 and 801 genes, which is a range similar to the gene counts from known complete TM7 genomes (e.g., the 754 genes of “*Ca*. Nanosynbacter lyticus” TM7x). Thus, we infer that these new genome assemblies are of similar quality to complete TM7 genomes. We excluded the TM7-033 genome from our study, since it was 100% identical to TM7-037 and was isolated from the same patient.

Phylogenetic analysis using GToTree (30) based on amino acid sequences of 60 concatenated core genes (Fig. 5B) improved upon the 16S rRNA phylogeny in that all nodes had bootstrap support above 0.5, likely due to the increase in signal afforded by the 60 genes as opposed to the 16S rRNA gene alone. Intriguingly, neither TM7-001 nor TM7-076 reinfected the ICM47 basibiont (Fig. 4A) despite differing in their relationship with JN023. However, the phylogenetic relationships estimated by the 16S tree do not explain the observed HMT-352/basibiont infection patterns, as similarly behaving strains, like TM7-001 and TM7-037, were shown to be distantly related.

To investigate genetic differences among the isolated HMT-352 strains and their possible correlates to symbiotic phenotypes, we compared the genetic composition of each genome (Fig. 5A). All gene sequences from each genome were clustered based on amino acid identity (AAI) to define 1,372 gene clusters, bins of putatively homologous genes. A total of 545 gene clusters were shared by all nine HMT-352 genomes, comprising 69 to 75% of each genome’s gene cluster content. Nearly all core gene clusters were present as a single copy in each genome (*N* = 534, 98%). These core genes encoded a number of conserved housekeeping functions, like ribosomal proteins, as well as other functions, like the arginine deiminase system and type IV secretion machinery (Table S5). The remaining 827 gene clusters contained genes contributed by one to eight of the genomes, with 442 gene clusters occurring in only a single genome (21 to 96 gene clusters unique to any given genome). Interestingly, no population structure in terms of shared gene content distinguished the TM7 strains based on their relationship to their hosts. Thus, while all HMT-352 strains share the majority of their genes, a given pair of strains could differ by over a hundred genes, allowing for substantial phenotypic variability.

10.1128/msystems.01488-21.9 TABLE S5 List of core genes that are common to all isolated HMT-352 strains. Download Table S5, XLSX file, 1.2 MB. Copyright © 2022 Nie et al. 2022 Nie et al. https://creativecommons.org/licenses/by/4.0/ This content is distributed under the terms of the Creative Commons Attribution 4.0 International license.

Pairwise average nucleotide identity (ANI) over alignable genome fractions supported this view of substantial intraspecies genetic diversity among HMT-352 strains (Fig. 5A, black-and-white heatmap). ANI values ranged between 93 and 95%, at or below the extreme end of the accepted range for intraspecific variation (31–33). Thus, the large number of genes shared by all nine genomes apparently harbor unexpectedly high nucleotide diversity, warranting further investigation into this strain-level genomic heterogeneity.

### Genomic variances correlate with phenotypic differences.

**FIG 6:**
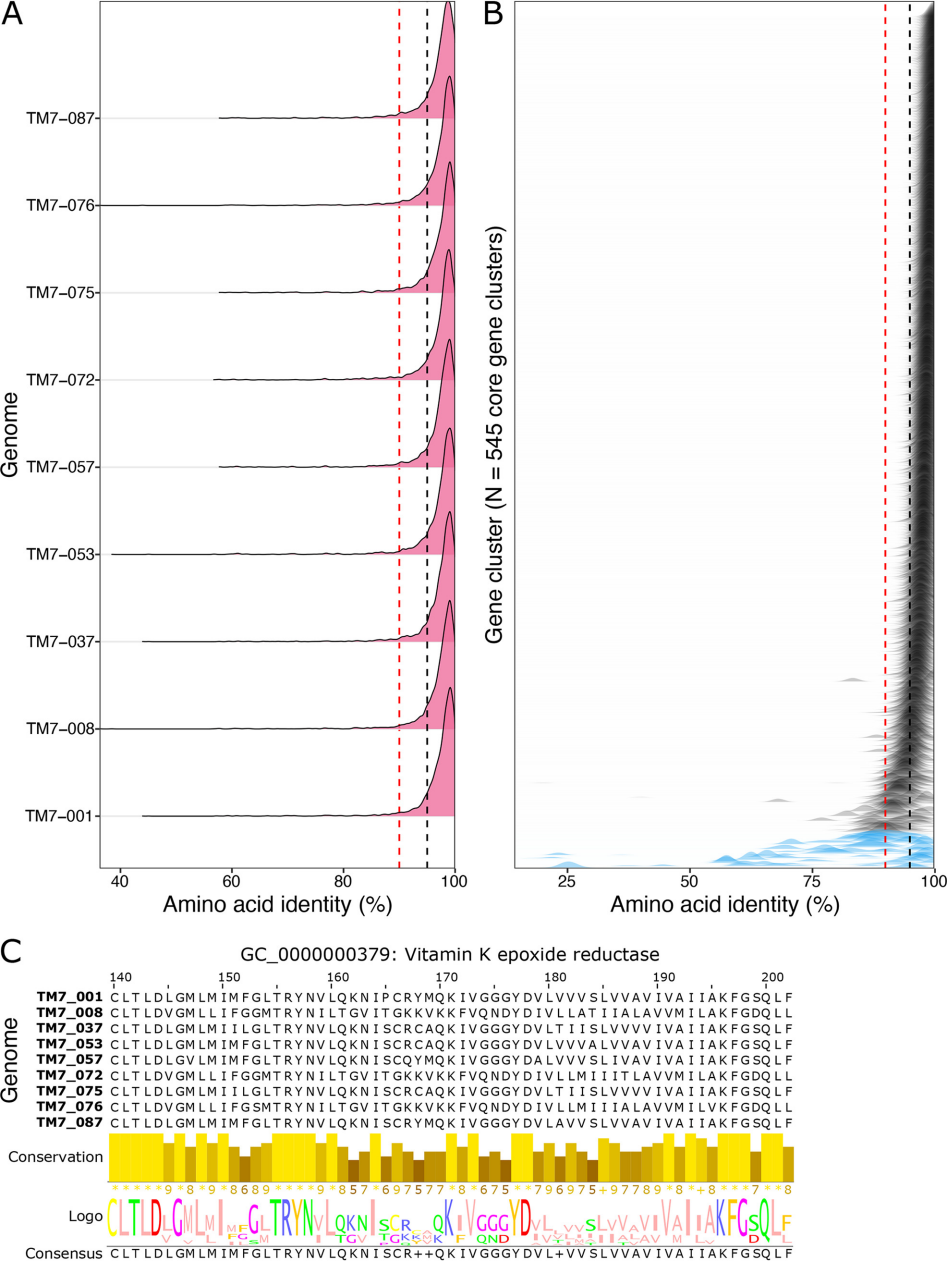
Pairwise amino acid identity for HMT-352 core genes. Sequence identity was computed by gene cluster for all pairwise combinations of gene sequences for all nine genomes. Black and red dotted lines mark the 95% and 90% thresholds. (A) Density plots of pairwise amino acid sequence identity (*x* axis) by genome. Each density plot summarizes the observed amino acid sequence similarities compared to the other 8 genomes. (B) Density of observed percent identities by gene cluster, ordered by mean percent identity. Each distribution represents a different gene cluster. (C) Alignment and logo plot of a representative window of the vitamin K epoxide reductase gene. The height and shading intensity of the bars below the alignment show the conservation of each residue across the different genomes, while the logo plot below represents the frequency of the observed residues by the letter size.

To further investigate this intraspecies genetic diversity, we compared the amino acid identity among the 545 gene clusters shared by all nine HMT-352 strains (Fig. 6). The majority of gene sequences for each of these gene clusters were above 95% similar across all genomes, and each genome’s distribution of amino acid identities was largely similar (Fig. 6A). This result supports the overall ANI conclusion that all genomes are approximately equally different genetically, i.e., no one strain is particularly deviant. However, the left shoulder of each genome’s distribution revealed a noticeable number of core genes with amino acid identities below 95% and even some below 90% identity (Fig. 6A, black and red dashed lines). Visualizing the amino acid identities by gene cluster (Fig. 6B) confirms the trend for the majority of shared genes but also reveals a small number of genes with wide ranging and often low amino acid percent identities. Indeed, 22 gene clusters had mean amino acid percent identities below 90% (blue-shaded distributions). Such highly variable gene sequences for core genes could explain the phenotypic variability within HMT-352, as this high intraspecific genetic variation encodes high amino acid differences, which in turn may engender differences in protein functionality.

We divided these data into the single-copy core gene clusters with a mean of less than 90% amino acid identity across the HMT-352 genomes and identified *N* = 17 single-copy core gene clusters. Of these, 6 could be annotated, encoding a protein secreted by the type IV secretion system, the tyrosine tRNA charger *tyrS*, an adenylate cyclase, a protein predicted to be involved in chromosome condensation, a vitamin K epoxide reductase, and a *cpoA* glycosyl transferase (Fig. 6C and Table S6). Sequence similarity patterns were largely distinct between each of these 17 gene clusters (Fig. S4), suggesting that these genes are not all coinherited and may not be linked to a single ecological function. Interestingly, the gene cluster GC_00000518, encoding an unannotated protein, was the only gene cluster among these to show a uniquely close phylogenetic relationship between TM7-001 and TM7-037, the only two strains that did not infect JN023. Potentially, this gene of unknown function may play a role in the observed phenotypic similarity between the two in terms of JN023 basibiont infection (Fig. 4A).

10.1128/msystems.01488-21.4 FIG S4 Heatmap of amino acid percent identities for gene sequences for the 17 core genes present in a single copy in each genome but with an average percent identity of less than 90%. Each subpanel is a different gene cluster, and the shading reports the percent identity between gene sequence pairs. All gene clusters consist of a single gene sequence per genome. Download FIG S4, PDF file, 0.05 MB. Copyright © 2022 Nie et al. 2022 Nie et al. https://creativecommons.org/licenses/by/4.0/ This content is distributed under the terms of the Creative Commons Attribution 4.0 International license.

10.1128/msystems.01488-21.10 TABLE S6 Six single-copy core gene clusters that can be annotated with a mean of less than 90% amino acid identity across the HMT-352 genomes (*N* = 17). Download Table S6, XLSX file, 0.01 MB. Copyright © 2022 Nie et al. 2022 Nie et al. https://creativecommons.org/licenses/by/4.0/ This content is distributed under the terms of the Creative Commons Attribution 4.0 International license.

## DISCUSSION

In previous studies, based on the characteristic features of TM7, such as their growth on the surface of specific host bacteria, an ultrasmall cell size, and the ability of free-floating cells to infect new host bacteria, the baiting method was developed and several TM7 species were isolated (4, 5). In this study, we expanded the baiting method and tested many host bacteria simultaneously. Compared to other isolation techniques, our method appears to be a more promising and straightforward way to test multiple host bacteria and saliva samples (could apply to other samples) in parallel to isolate TM7. Evidence from the baiting method implies that, when inoculated with a suitable host, even a very low abundance of free-floating TM7 cells can infect the host. This method also simplifies the previous baiting method by directly filtering the saliva sample without targeted enrichment or ultracentrifuging.

To date, all hosts of isolated TM7 are from the phylum *Actinobacteria* (3–5, 16). Non-*Actinobacteria*, including species from *Proteobacteria*, *Bacteroidetes*, *Firmicutes*, and *Fusobacteria*, were used to bait TM7 but were not susceptible to infection (4, 12). Non-*Actinobacteria* were also included in our preliminary host bank but failed to bait additional TM7 species. Previous literature in combination with our results imply that TM7 have a narrow host range, mainly restricted in *Actinobacteria*, compared to other predatory bacteria, such as *Bdellovibrio* and *Micavibrio*, which have taxonomically broad host ranges (34, 35). Identifying additional host bacteria in the future will be key in culturing currently uncultivated TM7 species. Even though our starting saliva sample contained many different species of TM7 (14), only particular groups of TM7 were isolated, suggesting that we need to test an even broader group of hosts both closely and distantly related to currently tested host bacteria. Furthermore, recently cultured environmental TM7 bacteria were found to have *Actinobacteria* hosts (10). However, *Absconditabacteria*, another phylum within the CPR, were recently found to associate with non-*Actinobacteria* hosts of the genus *Halochromatium* (*Gammaproteobacteria*, *Chromatiaceae*) (36). This observation raises several intriguing possibilities: (i) actinobacterial association may be a synapomorphy of TM7 relative to other CPR and (ii) non-actinobacterial hosts remain to be discovered for uncultivated TM7, especially from other divergent groups (14).

Our previous study demonstrated that, in relation to TM7, basibionts could either be completely resistant to TM7 infection or susceptible to TM7 infection (12). Here, however, we describe a new phenotypic response of bacterial hosts to TM7 infection, namely, low (LE) and high (HE) enrichment. In the LE group, the TM7 successfully cocultured with their host but at very low abundance, detectable by PCR but difficult to observe microscopically. Previously, decreased susceptibility of host XH001 to TM7x over time was observed, suggesting a rapid evolution gives rise to a long-term parasitic relationship in which both species persist. For the LE group, more study is needed to reveal the mechanism by which TM7 interact with their host and whether the symbiosis might evolve to be more favorable to the TM7. However, from our results we can conclude that the amount of TM7 associated with a particular basibiont can vary drastically depending on the phylogeny of the basibiont. This finding suggests there are specific receptors or docking proteins that vary in presence or expression across TM7 phylogeny. It is critical to point out that the observed phenotypes are all under laboratory *in vitro* conditions, and they may change according to the environmental niche in the oral cavity. For example, external forces such as nutrient availability, surrounding bacteria in the biofilm, and the immune system all can affect the phenotypic observations that we made. However, we believe that fundamental features such as selecting specific host bacteria do not change.

The genomic variation observed within the “*Ca*. Nanosynbacter sp.” strain HMT-352 presents a surprising contrast between their apparent similarity based on shared gene content yet dissimilarity based on ANI. Each of the HMT-352 strains shared a majority of its genome with all other studied HMT-352 strains, with approximately 70% of the genes in each genome being core to all HMT-352 strains. This proportion is similar to that of other studied bacterial species, although the proportion can vary substantially based on both the number of genome representatives and the degree of gene flow (37–39). However, despite the predicted homology for such large portions of the genome, the nucleotide sequences of the homologous regions differed substantially, resulting in ANI values between 93% and 95%. These ANI values stand out from typical intraspecific ANI values, which are at or above 95% or 97%, although known exceptions exist (31, 32, 40). One explanation for this apparent inconsistency between ANI and gene content could be that obligate symbionts have faster (41, 42) or variable (43) mutation rates. If so, then one must also invoke strong selection across a broad portion of the genome to prevent major additions or losses of genes. Altogether, the genomic variations revealed here within HMT-352 strains present an intriguing conundrum worthy of future investigation.

Host range is a fundamental feature of parasitic interactions. Compared to phages (44) and predatory bacteria such as *Bdellovibrio*, orally derived TM7 have a more narrow host range. Phylogenetic analysis showed that the TM7 host range pattern largely corresponds to the phylogenetic differences between basibionts only at broad levels (e.g., *Schaalia* clade 1 and *Actinomyces* clade 2). For example, HMT-352 and HMT-952 mostly infect clade 1 *Schaalia*, while HMT-957 mostly prefers clade 2 *Actinomyces*. However, little congruence could be found between the strain-level phylogeny of HMT-352 and their host range patterns. For example, within HMT-352 strains, the host range pattern is poorly correlated with *Schaalia* phylogeny. This result indicates that host range is not a predominantly vertically transmitted trait or perhaps that host range is a fast-evolving trait relative to the evolution of phylogenomic marker genes. Exploring these possibilities requires additional improvements to our current infection model, the model’s subsequent readout, and our TM7 strain-level collection.

Furthermore, using the most susceptible hosts found in this study, JN023 and ICM47, we tested host sensitivity to TM7 infection by assessing the TM7 ability to infect and induce the growth-crash phenomenon. The permissive hosts demonstrated the growth-crash phenotype, while the nonpermissive hosts did not display the growth-crash phenotype relative to specific TM7 strains (16). Interestingly, TM7 strains that did not infect these basibionts were not consistently clustered together phylogenetically or by gene content. For example, TM7-001 and TM7-037 were the only two strains that did not infect or induce the growth-crash phenotype in host bacteria, despite comparisons based on ANI, phylogenomic reconstruction, and gene content all concurring that these two strains were each more closely related to other strains than to each other. One possible interpretation of this phylogenetic-to-phenotypic discrepancy is to invoke plasticity in host association, the fact that one TM7 strain has many different but suitable *Schaalia* or *Actinomyces* hosts in the mouth, which led to the decoupling of genes related to host association from the rest of the TM7 genome. From this perspective, the predominant selective forces that shape oral TM7 speciation may be factors such as defense against phage. An alternative explanation involves horizontal gene transfer among different TM7, producing the observed result whereby host JN023 was sensitive to TM7-076 while ICM47 was not.

Here, we establish a high-throughput method for TM7 isolation. We effectively isolated many TM7 strains from the oral cavity. By exploring the phenotypic characteristics of these strains, we found that they could be divided into low-enrichment and high-enrichment groups, adding a third phenotype to the permissive and nonpermissive characterizations of TM7 infection. In this study, we examined strain variations of TM7 for the first time both phenotypically and genetically. TM7 strains from the same species showed diverse relationships with hosts. Most importantly, we demonstrated that strains with nearly identical genetic structure can have divergent ANI and AAI characteristics and display different host affinities. Future studies focused on investigating the potential gene functions that drive host bacterial responses in oral TM7 may eventually reveal the mechanisms governing the episymbiotic interaction between TM7 and their host bacteria.

## MATERIALS AND METHODS

### Bacterial growth conditions.

The oral *Actinobacteria* strains used in this study were cultured in brain heart infusion medium (BHI) at 37°C under microaerophilic (2.6% O_2_, 5% CO_2_, balanced with N_2_) or anaerobic (0% O_2_, 5% CO_2_, 5% H_2_, balanced with N_2_) conditions (see Table S1 in the supplemental material). The TM7 strains were cocultured under microaerophilic or anaerobic conditions with their basibionts (Table S2).

### Basibiont *Actinobacteria* isolation.

Unstimulated saliva samples were collected from 16 patients aged 18 years or older with stage I to IV periodontal disease (with equal sex representation). The study was done under the Forsyth IRB protocol number 21-02. The collected samples were centrifuged at 3,000 × *g* for 5 min, and the supernatant was collected and diluted 10^3^-, 10^4^-, and 10^5^-fold with BHI; 200-μL aliquots of the diluted samples were plated on three different *Actinobacteria*-selective agar plates (Table S3) and inoculated in 37°C microaerophilic and anaerobic chambers for 5 to 7 days. Three replicates were made for each concentration in each medium condition, with a total of 9 plates per medium condition. The *Actinobacteria*-selective agar was prepared based on previously published literature (Table S3) (45, 46). A single colony was picked under a stereomicroscope and replicate inoculated into two separate tubes containing BHI medium. One culture was incubated in a microaerophilic chamber, while the other was grown anaerobically. If both cultures grew, we kept only the microaerophilically grown basibiont. The taxonomy of *Actinomyces* and *Schaalia* species strains was validated by full-length 16S rRNA sequencing. The 16S rRNA was amplified by 27F and 1492R (Table S4) primers and sequenced by Sanger sequencing (Psomagen). Resulting sequences were searched by BLAST directly on the eHOMD database to determine taxonomy. All sequences had more than 98% percent identity to the closest eHOMD strains.

10.1128/msystems.01488-21.7 TABLE S3 Actinobacteria selective medium composition. Download Table S3, XLSX file, 0.01 MB. Copyright © 2022 Nie et al. 2022 Nie et al. https://creativecommons.org/licenses/by/4.0/ This content is distributed under the terms of the Creative Commons Attribution 4.0 International license.

10.1128/msystems.01488-21.8 TABLE S4 PCR primers used in this study with specific conditions. Download Table S4, XLSX file, 0.01 MB. Copyright © 2022 Nie et al. 2022 Nie et al. https://creativecommons.org/licenses/by/4.0/ This content is distributed under the terms of the Creative Commons Attribution 4.0 International license.

### TM7 isolation using putative basibionts.

Saliva samples (1 mL) from 16 patients with periodontitis were collected, vortexed vigorously for 5 min, and centrifuged at 3,000 × *g* for 5 min. The supernatant was filtered through a 0.45-μm filter, and the flowthrough was diluted to 5 mL with BRF medium composed of 50% BHI, 45% RPMI, and 5% fetal bovine serum (FBS) broth; 50 μL of dilutions was added to each well of a 96-well plate containing 200 μL of the basibiont culture, bringing the final volume of each well to 250 μL. To prepare the basibionts, we added 76 different basibionts (Table S1) into 76 wells at a final OD_600_ of 0.05 in 200 μL of BHI. The remaining 20 wells were filled with 200 μL of sterile medium as a control. These basibionts were replicated into 16 plates for 16 different saliva samples using a 96-channel pipettor. The 96-well plate was incubated in a 37°C microaerophilic or anaerobic chamber.

The cocultures were passaged at a dilution of 1:10 every 2 days into fresh BRF medium. The presence of TM7 was monitored by phase-contrast microscopy (Nikon Eclipse E400) and PCR (Table S4) at the 4th and 6th passages. If the cocultures were positive for TM7, 16S rRNA sequencing was performed to determine the taxonomy of the TM7. Again, all sequences had more than 98% identity to the closest eHOMD strain. After multiple passages, if we do get contamination from other oral bacteria, the majority of the time we detected *Campylobacteria* as a contaminant. However, by controlling the flow of the saliva through a 0.45-μm filter (i.e., filtering gently), we generally avoided contamination. If, however, contamination occurred and persisted after a few passages, we conducted the following procedures to clean the culture.

### Cleaning the TM7 cocultures.

The cultures were diluted 10^3^-, 10^4^-, and 10^5^-fold with BHI, and then 100 μL was plated on BHI blood agar and incubated for 5 to 7 days in an anaerobic or microaerophilic chamber (Table S1). Depending on the contamination level, how fast the contaminant grew, and at which passage we decided to plate the bacteria, we usually observed that 10 to 40% of the colonies had irregular morphology and tested positive for TM7. We typically tried to plate contaminated cultures early in passaging so that the contamination was minimal while still allowing enough time for the TM7 to enrich on the host bacteria. Ten to 20 colonies with irregular morphology were collected from each coculture and inoculated in 0.5 mL BRF medium. Passaging was performed every 24 h in BRF medium with a 1:10 dilution until the 4th or 6th passage (12). Growth conditions followed Table S1 unless specified otherwise. The presence of TM7 was confirmed by PCR using TM7 universal primers (Table S4, partial PCR primers) and phase-contrast microscopy. Cocultures that tested positive for TM7 by both PCR and phase-contrast microscopy were defined as the high-enrichment (HE) group. Cocultures that tested positive for TM7 using PCR but contained little to no detectable TM7 cells by phase-contrast microscopy were defined as the low-enrichment (LE) group. The taxa of purified TM7 and their basibionts were validated by full-length 16S rRNA sequencing as described above (Table S4).

### FISH staining.

FISH was carried out according to our previously described protocol (13). Briefly, cocultures were centrifuged and the pellets were resuspended in 1 mL of fixative solution (4% formaldehyde in 1× PBS, pH 7.4) for 3 h at room temperature. Fixed cells were centrifuged at 13,000 × *g* for 5 min, and the pellet was resuspended in 0.5 mL of 2-mg/mL lysozyme (100 or 20 mM Tris, 5 mM EDTA, pH 7; prepared 30 min before use) and incubated for 7 to 9 min at 37°C. Cells were pelleted and washed by resuspending each pellet in 0.5 mL of 50%, 80%, and 90% ethanol, with pelleting between each wash. For each pelleting step, centrifugation of 13,000 × *g* for 5 min was used. Finally, the cell pellets were dried in a speed vacuum dryer for 10 min.

For staining, cells were resuspended in 0.5 mL of hybridization solution (20 mM Tris-HCl, 0.9 M NaCl, 0.01% SDS, 30% deionized formamide, pH 8.0) and incubated at 37°C for 15 min. Fluorescently labeled oligonucleotide probes (TM7-567-Cy5, 5′-CCTACGCAACTCTTTACGCC-3′) were added to a final concentration of 5 ng/μL. The solution was incubated for 3 h at 42°C in the dark and centrifuged to collect the cell pellet. The pellet was washed twice by resuspension in 0.1 mL of 0.1× saline sodium citrate buffer (SSC; 1× SSC is 0.15 M NaCl plus 0.015 M sodium citrate) and incubated for 10 min at 37°C in the dark. For the last wash step, 5 μM (final concentration) Syto9 dye (1:1,000 diluted with SSC buffer; Invitrogen) was added to stain DNA of the basibiont bacteria. Cells were mounted on a PolyLys (19320-B; FisherScientific)-coated coverslip with 4 μL of SlowFade gold antifade reagent (Invitrogen). To coat the coverslip, we dipped the coverslip in PolyLys solution for 2 min, washed it with water, and dried it. The slides were visualized using a Zeiss 780 confocal microscope equipped with a 63×/1.4 Plan-Apochromat oil immersion objective (47). Images were processed by merging the two-color channels in FIJI, a distribution of imageJ2, without changing the pixel intensity (48).

### qPCR quantification of TM7.

The genomic DNA (gDNA) of each coculture was isolated using the MasterPure Complete DNA purification kit (Lucingen; Epicentre) by following the manufacturer’s guidelines, and its concentration was determined using Qubit (Invitrogen). The gDNA was diluted to 0.1, 0.01, 0.001, 0.0001, and 0.00001 ng/μL for both the DNA template standard and the bacterial coculture. A volume of 4.5 μL of gDNA from each dilution was mixed with 5 μL PowerUp SYBR green master mix and 0.25 μL of the appropriate forward and reverse quantitative reverse transcription-PCR primers (AJ13-F, 5′-GTGACTGGGCGTAAAGAGTT-3′; AJ14-R, 5′-CTACGGATTTCACTCCTAC-3′). For TM7, the reactions were processed at an initial denaturation temperature of 95°C for 1 min, followed by a 50-cycle amplification step, which consisted of denaturation at 95°C for 30 s, annealing at 54°C for 30 s, and extension at 72°C for 30 s. A standard curve was generated using a plasmid containing the 16S sequence of the TM7 bacteria. Using the generated standard curve, the coculture gDNA TM7 16S copy number was determined.

### Host range mapping of TM7 isolates.

The cocultures of TM7 strains from the HE group were reinoculated in BRF medium and passaged every 24 h for 2 to 4 passages. Due to a small number of isolates, we did not test the LE group. Each coculture was filtered through a 0.45-μm filter, and 25 μL of the flowthrough was added to 175 μL of 0.05 OD_600_ candidate host inoculum in BRF medium; 2.3 mL of fresh BRF medium was added to each mixed broth, bringing the final volume to 2.5 mL. The infection experiments were incubated in a microaerophilic chamber at 37°C for 24 h. The cocultures were passaged into 4 mL of fresh medium with a final OD_600_ of 0.1. The cocultures were incubated for 24 h and passaged again to an OD_600_ of 0.1. This continued until the 6th passage. To determine if TM7 infection occurred and remained stable, the presence of TM7 was validated by PCR at the 4th and 6th passages.

### Genome sequencing and assembly.

The TM7 coculture was reinoculated as described above and expanded into 200 mL of BHI culture. The coculture was then centrifuged at 3,500 × *g*, and the supernatant containing free-floating TM7 was filtered through a 0.45-μm Stericup (Millipore). The flowthrough was ultracentrifuged at 80,000 × *g* for 80 min. The pellets were resuspended in 1 mL of fresh BHI. The purity of the isolated TM7 cells was confirmed by phase-contrast microscopy, and, using the EpiCenter Gram-positive DNA isolation kit, the TM7 gDNA was extracted from both the filtration-isolated TM7 and the original coculture to produce two genomic libraries per TM7 strain.

gDNA was randomly fragmented by sonication, and then the gDNA fragments were end polished, A-tailed, and ligated with the full-length adapters of Illumina sequencing. Further PCR amplification was then performed with P5 and indexed P7 oligonucleotides. The PCR products, as the final construction of the libraries, were purified with the AMPure XP system. Libraries were checked for size distribution by an Agilent 2100 Bioanalyzer (Agilent Technologies, CA, USA) and quantified by real-time PCR to pool libraries at a final concentration of 3 nM DNA. The libraries were then sequenced on an Illumina Novaseq (PE; 150-bp reads).

All code used to assemble and analyze the genomes is available in a reproducible narrative methods document at https://www.borlab.org/resources. For each new TM7 strain, genomes were assembled using anvi’o (49) through a two-step process based on metaspades v3.15.3 (50) and MaxBin2 (51). Briefly, libraries from the isolated TM7 were first individually assembled with metaspades, resulting in 9 separate assemblies for the 9 TM7. The short reads from the isolate and coculture libraries for each TM7 strain then were mapped back to that strain’s assembly with bowtie2 (52). MaxBin2 then used this mapping information to automatically bin assembled contiguous sequences (contigs) into draft genome bins on the basis of read mapping and contig tetranucleotide frequency. MaxBin2 bins were manually refined in anvi’o, and contigs from the refined TM7 bins were then exported. For each strain, metaspades was then rerun as a coassembly using both the isolate and coculture libraries and incorporating the previously refined TM7 contigs via the –untrusted-contigs flag, which uses the supplied contigs to improve gap closure and repeat resolution but not for graph construction. Read mapping, automated binning, and manual bin refinement were then repeated as described before to generate the final genome bins. Genes were called with prodigal (53) and annotated with eggNOG-mapper v2 (54).

### Pangenome and bioinformatic analysis.

The newly assembled TM7 genomes were used to construct a pangenome, the set of all genes known from a group of organisms, with anvi’o (49). Briefly, amino acid sequences for all genes were compared against each other using BLASTP (55), and these similarities were then clustered with the Markov clustering algorithm (56). The output of this analysis is termed gene clusters, as they are sets of gene sequences clustered based on sequence similarity. Thus, each gene cluster represents a distinct, putatively homologous gene.

A phylogenomic tree was constructed with GToTree (30) using a concatenated set of amino acid sequences for 60 bacterial single-copy core genes. Briefly, GToTree uses prodigal (53) to call genes, HMMER (57) to identify the target single-copy core genes, muscle and TrimAl to align and trim each gene (58, 59), and FastTree2 to generate an approximately maximum-likelihood tree (60). The “*Candidatus* Nanosynbacter lyticus” TM7x genome (HMT-952) was downloaded from NCBI and used as an outgroup. The resultant tree was visualized with FigTree.

Average nucleotide identity (ANI) was computed between all genome pairs in the anvi’o pangenome using BLASTN (55) via pyani (61). For visualization, only the ANI over the aligned fraction was used, as total ANI could be sensitive to small methodological variations in assembly size.

Amino acid similarities among gene sequences for core gene clusters were calculated by exporting sequence alignments for all core genes using the anvi-get-sequences-for-gene-clusters command (4,946 gene sequences representing 545 gene clusters). The resultant fasta was then parsed using a custom R script (narrative methods) to obtain percent identities between all pairwise combinations of gene sequences for each gene cluster. Percent identities were calculated in R using the seqinr package.

### Data availability.

The genomes were deposited in GenBank with the accession numbers JAJQJX000000000, CP089290, JAJQJW000000000, JAJQJV000000000, CP089289, JAJQJU000000000, JAJQJT000000000, JAJQJS000000000000, CP089288, and JAJQJR000000000 under BioProject no. PRJNA784561.

## References

[1] Abusleme L, Dupuy AK, Dutzan N, Silva N, Burleson JA, Strausbaugh LD, Gamonal J, Diaz PI. 2013. The subgingival microbiome in health and periodontitis and its relationship with community biomass and inflammation. ISME J 7:1016–1025. doi:10.1038/ismej.2012.174. 23303375 PMC3635234

[2] Griffen AL, Beall CJ, Campbell JH, Firestone ND, Kumar PS, Yang ZK, Podar M, Leys EJ. 2012. Distinct and complex bacterial profiles in human periodontitis and health revealed by 16S pyrosequencing. ISME J 6:1176–1185. doi:10.1038/ismej.2011.191. 22170420 PMC3358035

[3] Cross KL, Campbell JH, Balachandran M, Campbell AG, Cooper SJ, Griffen A, Heaton M, Joshi S, Klingeman D, Leys E, Yang Z, Parks JM, Podar M. 2019. Targeted isolation and cultivation of uncultivated bacteria by reverse genomics. Nat Biotechnol 37:1314–1321. doi:10.1038/s41587-019-0260-6. 31570900 PMC6858544

[4] Bor B, Collins AJ, Murugkar PP, Balasubramanian S, To TT, Hendrickson EL, Bedree JK, Bidlack FB, Johnston CD, Shi W, McLean JS, He X, Dewhirst FE. 2020. Insights obtained by culturing Saccharibacteria with their bacterial hosts. J Dent Res 99:e002203452090579. doi:10.1177/0022034520905792. PMC7243422 32075512

[5] Murugkar PP, Collins AJ, Chen T, Dewhirst FE. 2020. Isolation and cultivation of candidate phyla radiation *Saccharibacteria* (TM7) bacteria in coculture with bacterial hosts. J Oral Microbiol 12:1814666. doi:10.1080/20002297.2020.1814666. 33209205 PMC7651992

[6] Escapa IF, Chen T, Huang Y, Gajare P, Dewhirst FE, Lemon KP. 2018. New insights into human nostril microbiome from the expanded human oral microbiome database (eHOMD): a resource for the microbiome of the human aerodigestive tract. mSystems 3:e00187-18. doi:10.1128/mSystems.00187-18. PMC6280432 30534599

[7] Castelle CJ, Banfield JF. 2018. Major new microbial groups expand diversity and alter our understanding of the tree of life. Cell 172:1181–1197. doi:10.1016/j.cell.2018.02.016. 29522741

[8] Hug LA, Baker BJ, Anantharaman K, Brown CT, Probst AJ, Castelle CJ, Butterfield CN, Hernsdorf AW, Amano Y, Ise K, Suzuki Y, Dudek N, Relman DA, Finstad KM, Amundson R, Thomas BC, Banfield JF. 2016. A new view of the tree of life. Nat Microbiol 1:16048. doi:10.1038/nmicrobiol.2016.48. 27572647

[9] Bor B, Bedree JK, Shi W, McLean JS, He X. 2019. Saccharibacteria (TM7) in the human oral microbiome. J Dent Res 98:500–509. doi:10.1177/0022034519831671. 30894042 PMC6481004

[10] Batinovic S, Rose JJA, Ratcliffe J, Seviour RJ, Petrovski S. 2021. Cocultivation of an ultrasmall environmental parasitic bacterium with lytic ability against bacteria associated with wastewater foams. Nat Microbiol 6:703–711. doi:10.1038/s41564-021-00892-1. 33927381

[11] Xie B, Wang J, Nie Y, Chen D, Hu B, Wu X, Du W. 2021. EpicPCR-directed cultivation of a Candidatus Saccharibacteria symbiont reveals a type IV pili-dependent epibiotic lifestyle. bioRxiv doi:10.1101/2021.07.08.451036.

[12] Bor B, McLean JS, Foster KR, Cen L, To TT, Serrato-Guillen A, Dewhirst FE, Shi W, He X. 2018. Rapid evolution of decreased host susceptibility drives a stable relationship between ultrasmall parasite TM7x and its bacterial host. Proc Natl Acad Sci USA 115:12277–12282. doi:10.1073/pnas.1810625115. 30442671 PMC6275545

[13] Bor B, Poweleit N, Bois JS, Cen L, Bedree JK, Zhou ZH, Gunsalus RP, Lux R, McLean JS, He X, Shi W. 2016. Phenotypic and physiological characterization of the epibiotic interaction between TM7x and its basibiont Actinomyces. Microb Ecol 71:243–255. doi:10.1007/s00248-015-0711-7. 26597961 PMC4688200

[14] McLean JS, Bor B, Kerns KA, Liu Q, To TT, Solden L, Hendrickson EL, Wrighton K, Shi W, He X. 2020. Acquisition and adaptation of ultra-small parasitic reduced genome bacteria to mammalian hosts. Cell Rep 32:107939. doi:10.1016/j.celrep.2020.107939. 32698001 PMC7427843

[15] Nouioui I, Carro L, García-López M, Meier-Kolthoff JP, Woyke T, Kyrpides NC, Pukall R, Klenk H-P, Goodfellow M, Göker M. 2018. Genome-based taxonomic classification of the phylum Actinobacteria. Front Microbiol 9:2007. doi:10.3389/fmicb.2018.02007. 30186281 PMC6113628

[16] Utter DR, He X, Cavanaugh CM, McLean JS, Bor B. 2020. The saccharibacterium TM7x elicits differential responses across its host range. ISME J 14:3054–3067. doi:10.1038/s41396-020-00736-6. 32839546 PMC7784981

[17] Baker JL, Bor B, Agnello M, Shi W, He X. 2017. Ecology of the oral microbiome: beyond bacteria. Trends Microbiol 25:362–374. doi:10.1016/j.tim.2016.12.012. 28089325 PMC5687246

[18] He X, McLean JS, Edlund A, Yooseph S, Hall AP, Liu S-Y, Dorrestein PC, Esquenazi E, Hunter RC, Cheng G, Nelson KE, Lux R, Shi W. 2015. Cultivation of a human-associated TM7 phylotype reveals a reduced genome and epibiotic parasitic lifestyle. Proc Natl Acad Sci USA 112:244–249. doi:10.1073/pnas.1419038112. 25535390 PMC4291631

[19] Duar RM, Lin XB, Zheng J, Martino ME, Grenier T, Pérez-Muñoz ME, Leulier F, Gänzle M, Walter J. 2017. Lifestyles in transition: evolution and natural history of the genus Lactobacillus. FEMS Microbiol Rev 41:S27–S48. doi:10.1093/femsre/fux030. 28673043

[20] Igboin CO, Griffen AL, Leys EJ. 2009. Porphyromonas gingivalis strain diversity. J Clin Microbiol 47:3073–3081. doi:10.1128/JCM.00569-09. 19675220 PMC2756895

[21] Bujold AR, Lani NR, Sanz MG. 2019. Strain-to-strain variation of Rhodococcus equi growth and biofilm formation in vitro. BMC Res Notes 12:519. doi:10.1186/s13104-019-4560-1. 31426832 PMC6701102

[22] Stevenson K. 2015. Genetic diversity of Mycobacterium avium subspecies paratuberculosis and the influence of strain type on infection and pathogenesis: a review. Vet Res 46:64. doi:10.1186/s13567-015-0203-2. 26092160 PMC4473831

[23] Amano A, Kuboniwa M, Nakagawa I, Akiyama S, Morisaki I, Hamada S. 2000. Prevalence of specific genotypes of Porphyromonas gingivalis fimA and periodontal health status. J Dent Res 79:1664–1668. doi:10.1177/00220345000790090501. 11023261

[24] Bongrand C, Ruby EG. 2019. The impact of Vibrio fischeri strain variation on host colonization. Curr Opin Microbiol 50:15–19. doi:10.1016/j.mib.2019.09.002. 31593868 PMC6899189

[25] Coats SR, Kantrong N, To TT, Jain S, Genco CA, McLean JS, Darveau RP. 2019. The distinct immune-stimulatory capacities of Porphyromonas gingivalis strains 381 and ATCC 33277 are determined by the fimB allele and gingipain activity. Infect Immun 87:e00319-19. doi:10.1128/IAI.00319-19. 31570556 PMC6867864

[26] Biswas S, Keightley A, Biswas I. 2019. Characterization of a stress tolerance-defective mutant of Lactobacillus rhamnosus LRB. Mol Oral Microbiol 34:153–167. doi:10.1111/omi.12262. 31056830 PMC6688488

[27] Lamont EI, Hendrickson EL, McLean JS, He X, Bor B. 2020. Complete genome sequence of strain BB001, a novel epibiont bacterium from the candidate phylum Saccharibacteria (TM7). Microbiol Resour Announc 9:e00810-20. doi:10.1128/MRA.00810-20. 32816985 PMC7441243

[28] Brown CT, Hug LA, Thomas BC, Sharon I, Castelle CJ, Singh A, Wilkins MJ, Wrighton KC, Williams KH, Banfield JF. 2015. Unusual biology across a group comprising more than 15% of domain Bacteria. Nature 523:208–211. doi:10.1038/nature14486. 26083755

[29] Castelle CJ, Brown CT, Anantharaman K, Probst AJ, Huang RH, Banfield JF. 2018. Biosynthetic capacity, metabolic variety and unusual biology in the CPR and DPANN radiations. Nat Rev Microbiol 16:629–645. doi:10.1038/s41579-018-0076-2. 30181663

[30] Lee MD. 2019. GToTree: a user-friendly workflow for phylogenomics. Bioinformatics 35:4162–4164. doi:10.1093/bioinformatics/btz188. 30865266 PMC6792077

[31] Jain C, Rodriguez-R LM, Phillippy AM, Konstantinidis KT, Aluru S. 2018. High throughput ANI analysis of 90K prokaryotic genomes reveals clear species boundaries. Nat Commun 9:5114. doi:10.1038/s41467-018-07641-9. 30504855 PMC6269478

[32] Olm MR, Crits-Christoph A, Diamond S, Lavy A, Matheus Carnevali PB, Banfield JF. 2020. Consistent metagenome-derived metrics verify and delineate bacterial species boundaries. mSystems 5:e00731-19. doi:10.1128/mSystems.00731-19. 31937678 PMC6967389

[33] Rodriguez-R LM, Konstantinidis KT. 2014. Bypassing cultivation to identify bacterial species: culture-independent genomic approaches identify credibly distinct clusters, avoid cultivation bias, and provide true insights into microbial species. Microbe Magazine 9:111–118. doi:10.1128/microbe.9.111.1.

[34] Dashiff A, Junka RA, Libera M, Kadouri DE. 2011. Predation of human pathogens by the predatory bacteria Micavibrio aeruginosavorus and Bdellovibrio bacteriovorus. J Appl Microbiol 110:431–444. doi:10.1111/j.1365-2672.2010.04900.x. 21114596

[35] Kadouri D, Venzon NC, O'Toole GA. 2007. Vulnerability of pathogenic biofilms to Micavibrio aeruginosavorus. Appl Environ Microbiol 73:605–614. doi:10.1128/AEM.01893-06. 17098913 PMC1796979

[36] Moreira D, Zivanovic Y, López-Archilla AI, Iniesto M, López-García P. 2021. Reductive evolution and unique predatory mode in the CPR bacterium Vampirococcus lugosii. Nat Commun 12:2454. doi:10.1038/s41467-021-22762-4. 33911080 PMC8080830

[37] Utter DR, Borisy GG, Eren AM, Cavanaugh CM, Mark Welch JL. 2020. Metapangenomics of the oral microbiome provides insights into habitat adaptation and cultivar diversity. Genome Biol 21:293. doi:10.1186/s13059-020-02200-2. 33323129 PMC7739467

[38] Cross KL, Leigh BA, Hatmaker EA, Mikaelyan A, Miller AK, Bordenstein SR. 2021. Genomes of gut bacteria from *Nasonia* wasps shed light on phylosymbiosis and microbe-assisted hybrid breakdown. mSystems 6:e01342-20. doi:10.1128/mSystems.01342-20. 33824199 PMC8547009

[39] Shaiber A, Willis AD, Delmont TO, Roux S, Chen L-X, Schmid AC, Yousef M, Watson AR, Lolans K, Esen ÖC, Lee STM, Downey N, Morrison HG, Dewhirst FE, Mark Welch JL, Eren AM. 2020. Functional and genetic markers of niche partitioning among enigmatic members of the human oral microbiome. Genome Biol 21:292. doi:10.1186/s13059-020-02195-w. 33323122 PMC7739484

[40] Goris J, Konstantinidis KT, Klappenbach JA, Coenye T, Vandamme P, Tiedje JM. 2007. DNA-DNA hybridization values and their relationship to whole-genome sequence similarities. Int J Syst Evol Microbiol 57:81–91. doi:10.1099/ijs.0.64483-0. 17220447

[41] Itoh T, Martin W, Nei M. 2002. Acceleration of genomic evolution caused by enhanced mutation rate in endocellular symbionts. Proc Natl Acad Sci USA 99:12944–12948. doi:10.1073/pnas.192449699. 12235368 PMC130565

[42] Moran NA. 1996. Accelerated evolution and Muller’s rachet in endosymbiotic bacteria. Proc Natl Acad Sci USA 93:2873–2878. doi:10.1073/pnas.93.7.2873. 8610134 PMC39726

[43] Boscaro V, Kolisko M, Felletti M, Vannini C, Lynn DH, Keeling PJ. 2017. Parallel genome reduction in symbionts descended from closely related free-living bacteria. Nat Ecol Evol 1:1160–1167. doi:10.1038/s41559-017-0237-0. 29046583

[44] de Jonge PA, Nobrega FL, Brouns SJJ, Dutilh BE. 2019. Molecular and evolutionary determinants of bacteriophage host range. Trends Microbiol 27:51–63. doi:10.1016/j.tim.2018.08.006. 30181062

[45] Tanner ACR, Mathney JMJ, Kent RL, Chalmers NI, Hughes CV, Loo CY, Pradhan N, Kanasi E, Hwang J, Dahlan MA, Papadopolou E, Dewhirst FE. 2011. Cultivable anaerobic microbiota of severe early childhood caries. J Clin Microbiol 49:1464–1474. doi:10.1128/JCM.02427-10. 21289150 PMC3122858

[46] Lewis R, McKenzie D, Bagg J, Dickie A. 1995. Experience with a novel selective medium for isolation of Actinomyces spp. from medical and dental specimens. J Clin Microbiol 33:1613–1616. doi:10.1128/jcm.33.6.1613-1616.1995. 7650197 PMC228226

[47] Montero Llopis P, Senft RA, Ross-Elliott TJ, Stephansky R, Keeley DP, Koshar P, Marqués G, Gao Y-S, Carlson BR, Pengo T, Sanders MA, Cameron LA, Itano MS. 2021. Best practices and tools for reporting reproducible fluorescence microscopy methods. Nat Methods 18:1463–1476. doi:10.1038/s41592-021-01156-w. 34099930

[48] Schindelin J, Arganda-Carreras I, Frise E, Kaynig V, Longair M, Pietzsch T, Preibisch S, Rueden C, Saalfeld S, Schmid B, Tinevez J-Y, White DJ, Hartenstein V, Eliceiri K, Tomancak P, Cardona A. 2012. Fiji: an open-source platform for biological-image analysis. Nat Methods 9:676–682. doi:10.1038/nmeth.2019. 22743772 PMC3855844

[49] Eren AM, Esen ÖC, Quince C, Vineis JH, Morrison HG, Sogin ML, Delmont TO. 2015. Anvi’o: an advanced analysis and visualization platform for ’omics data. PeerJ 3:e1319. doi:10.7717/peerj.1319. 26500826 PMC4614810

[50] Nurk S, Meleshko D, Korobeynikov A, Pevzner PA. 2017. metaSPAdes: a new versatile metagenomic assembler. Genome Res 27:824–834. doi:10.1101/gr.213959.116. 28298430 PMC5411777

[51] Wu Y-W, Simmons BA, Singer SW. 2016. MaxBin 2.0: an automated binning algorithm to recover genomes from multiple metagenomic datasets. Bioinformatics 32:605–607. doi:10.1093/bioinformatics/btv638. 26515820

[52] Langmead B, Salzberg SL. 2012. Fast gapped-read alignment with Bowtie 2. Nat Methods 9:357–359. doi:10.1038/nmeth.1923. 22388286 PMC3322381

[53] Hyatt D, Chen G-L, LoCascio PF, Land ML, Larimer FW, Hauser LJ. 2010. Prodigal: prokaryotic gene recognition and translation initiation site identification. BMC Bioinformatics 11:119. doi:10.1186/1471-2105-11-119. 20211023 PMC2848648

[54] Cantalapiedra CP, Hernández-Plaza A, Letunic I, Bork P, Huerta-Cepas J. 2021. eggNOG-mapper v2: functional annotation, orthology assignments, and domain prediction at the metagenomic scale. Bioinformatics 38:5825–5829. doi:10.1093/molbev/msab293. PMC8662613 34597405

[55] Altschul SF, Gish W, Miller W, Myers EW, Lipman DJ. 1990. Basic local alignment search tool. J Mol Biol 215:403–410. doi:10.1016/S0022-2836(05)80360-2. 2231712

[56] van Dongen S, Abreu-Goodger C. 2012. Using MCL to extract clusters from networks, p 281–295. *In* van Helden J, Toussaint A, Thieffry D (ed), Bacterial molecular networks. Springer New York, New York, NY. 10.1007/978-1-61779-361-5_15 22144159

[57] Eddy SR. 2011. Accelerated profile HMM searches. PLoS Comput Biol 7:e1002195. doi:10.1371/journal.pcbi.1002195. 22039361 PMC3197634

[58] Edgar RC. 2004. MUSCLE: multiple sequence alignment with high accuracy and high throughput. Nucleic Acids Res 32:1792–1797. doi:10.1093/nar/gkh340. 15034147 PMC390337

[59] Capella-Gutierrez S, Silla-Martinez JM, Gabaldon T. 2009. trimAl: a tool for automated alignment trimming in large-scale phylogenetic analyses. Bioinformatics 25:1972–1973. doi:10.1093/bioinformatics/btp348. 19505945 PMC2712344

[60] Price MN, Dehal PS, Arkin AP. 2010. FastTree 2–approximately maximum-likelihood trees for large alignments. PLoS One 5:e9490. doi:10.1371/journal.pone.0009490. 20224823 PMC2835736

[61] Pritchard L, Glover RH, Humphris S, Elphinstone JG, Toth IK. 2016. Genomics and taxonomy in diagnostics for food security: soft-rotting enterobacterial plant pathogens. Anal Methods 8:12–24. doi:10.1039/C5AY02550H.

