# Supplementary material for: Strain-Level Variation and Diverse Host Bacterial Responses in Episymbiotic Saccharibacteria
Source: mSystems. 2022 Mar 28;7(2):e01488-21. doi: 10.1128/msystems.01488-21 (PMC9040727; doi:10.1128/msystems.01488-21)
Supplement: FIG S1 [file msystems.01488-21-sf001.pdf]

Figure S1. Low enriched TM7 bacteria and their basibionts (phase and FISH images)

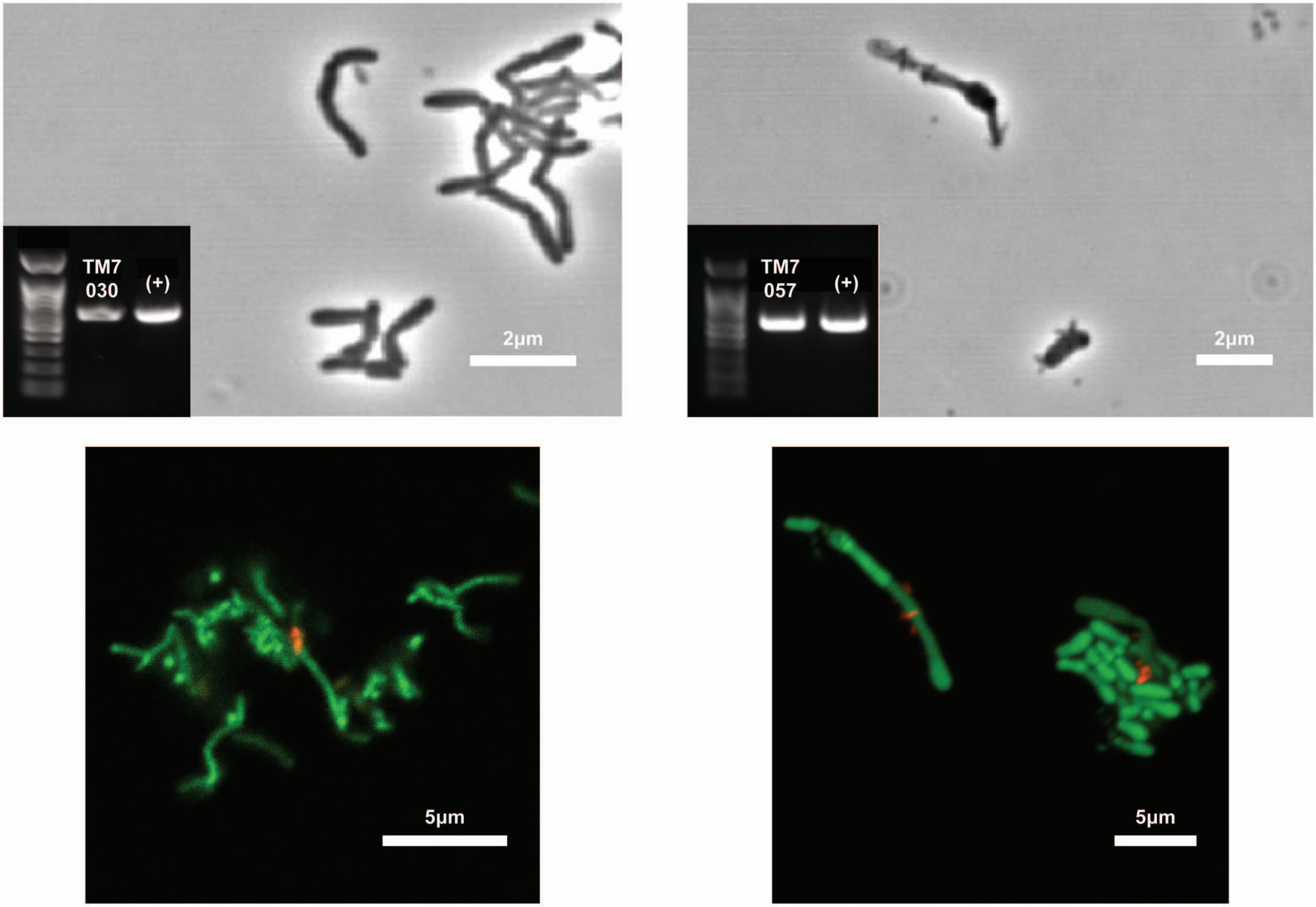

Low enriched TM7

High enriched TM7
