# Supplementary figures and images for: Strain-Level Variation and Diverse Host Bacterial Responses in Episymbiotic Saccharibacteria
Source: mSystems. 2022 Mar 28;7(2):e01488-21. doi: 10.1128/msystems.01488-21 (PMC9040727; doi:10.1128/msystems.01488-21)

Figure S2.

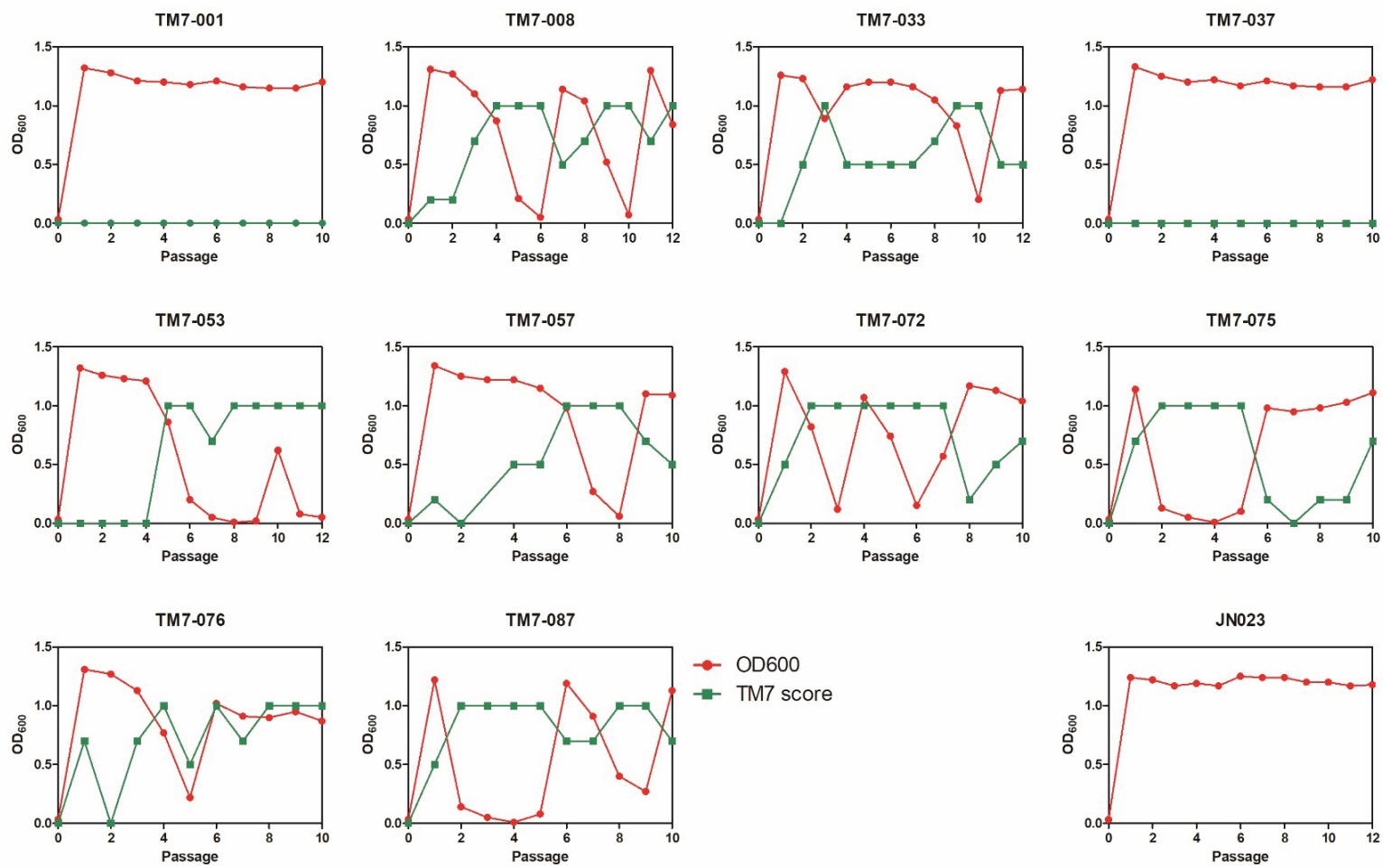

Supplement: FIG S2 [file msystems.01488-21-sf002.pdf]

Figure S3.

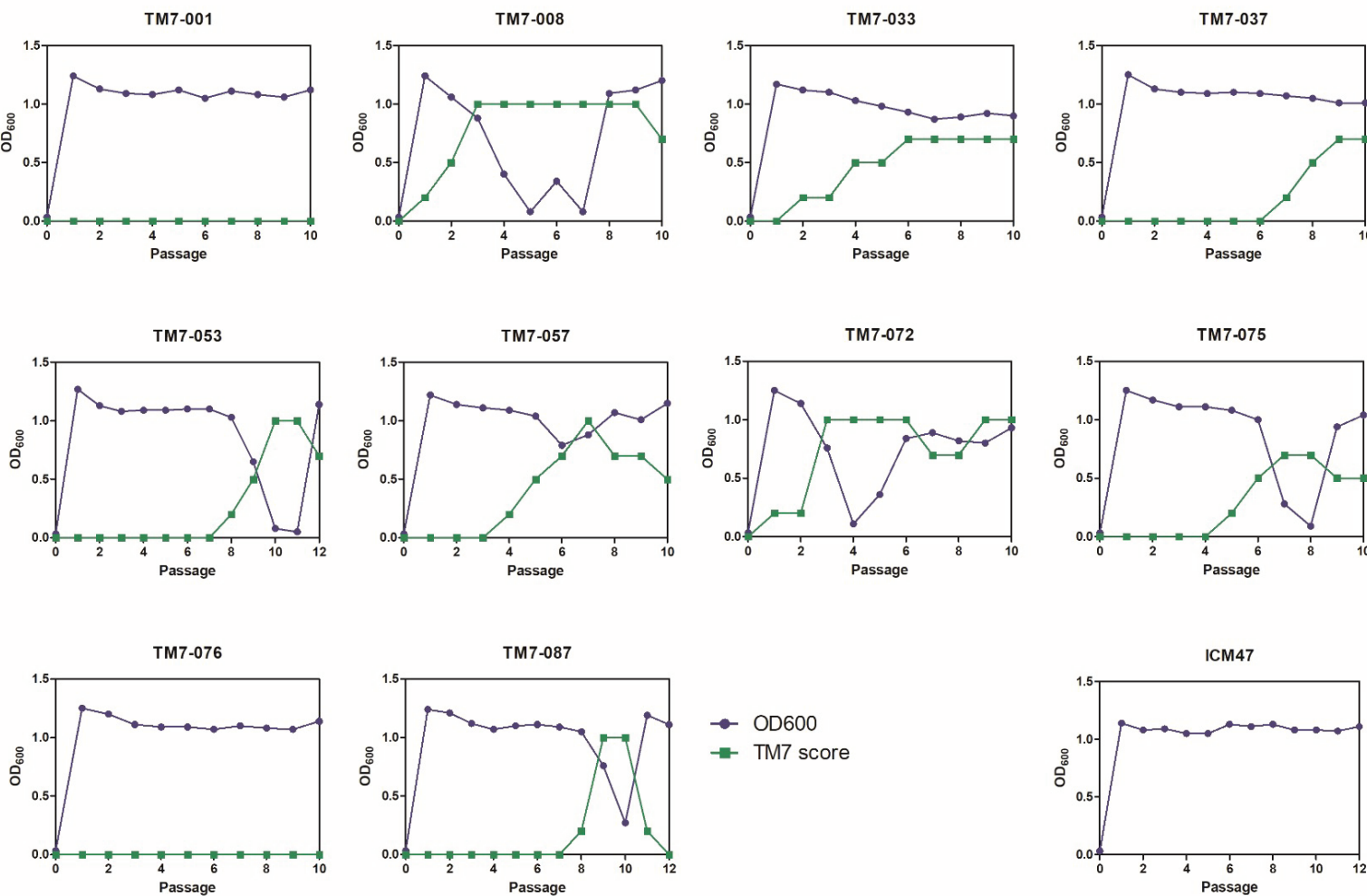

Supplement: FIG S3 [file msystems.01488-21-sf003.pdf]

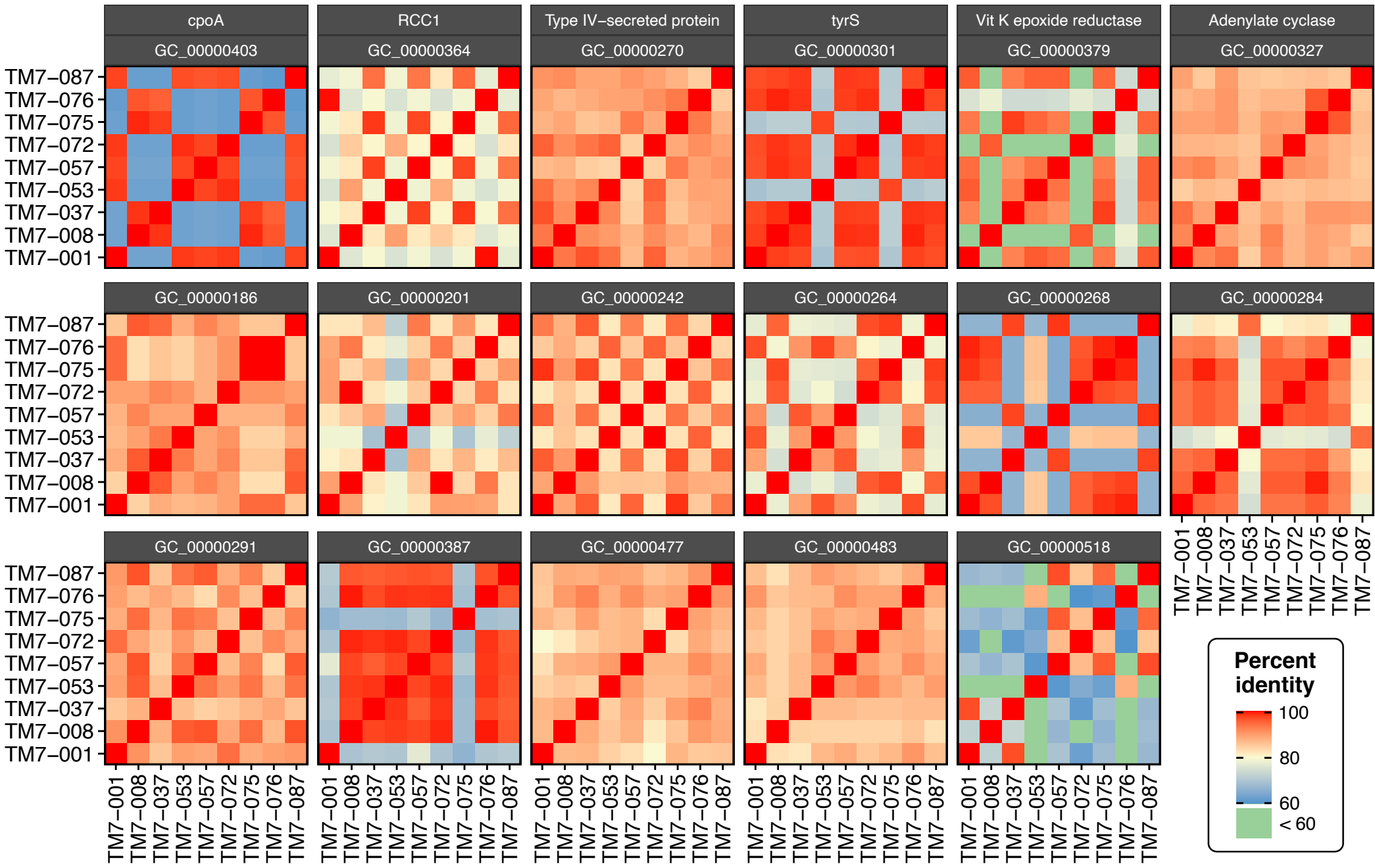

Supplement: FIG S4 [file msystems.01488-21-sf004.pdf]
